# Fibroblast growth factor-23 and the risk of cardiovascular diseases and mortality in the general population: A systematic review and dose-response meta-analysis

**DOI:** 10.3389/fcvm.2022.989574

**Published:** 2022-11-03

**Authors:** Menglu Liu, Panpan Xia, Ziqi Tan, Tiangang Song, Kaibo Mei, Jingfeng Wang, Jianyong Ma, Yuan Jiang, Jing Zhang, Yujie Zhao, Peng Yu, Xiao Liu

**Affiliations:** ^1^Department of Cardiology, Seventh People’s Hospital of Zhengzhou, Zhengzhou, China; ^2^Department of Endocrine, The Second Affiliated Hospital of Nanchang University, Nanchang, China; ^3^Department of Anesthesiology, People’s Hospital of Shangrao, Shangrao, China; ^4^Department of Cardiology, Sun Yat-sen Memorial Hospital, Sun Yat-sen University, Guangzhou, Guangdong, China; ^5^Department of Pharmacology and Systems Physiology, University of Cincinnati College of Medicine, Cincinnati, OH, United Status; ^6^Department of Anesthesiology, The Second Affiliated Hospital of Nanchang University, Nanchang, China

**Keywords:** FGF23, cardiovascular diseases, myocardial infarction, stroke, heart failure, mortality, meta-analysis

## Abstract

**Background:**

In the past decade, fibroblast growth factor 23 (FGF23) has been recognized as an important biomarker of cardiovascular diseases. This study aimed to assess the relationship between FGF23 and the risk of cardiovascular diseases (CVDs) in general populations.

**Methods:**

The protocol was registered prospectively in PROSPERO (CRD42021281837) and two authors independently searched for relevant studies in the PubMed, EMBASE, and Cochrane Library databases. The random effects model was applied.

**Results:**

In total, 29 prospective studies involving 135,576 participants were included. In the general population, the category analysis revealed that elevated FGF23 levels were related to increased risks of myocardial infarction (MI) (*RR*: 1.40, 95%*CI*: 1.03−1.89), stroke (*RR:* 1.20, 95%*CI:* 1.02−1.43), heart failure (HF) (*RR:* 1.37, 95%*CI:* 1.23−1.52), CVD events (*RR:* 1.22, 95%*CI:* 0.99−1.51), cardiovascular mortality (*RR:* 1.46, 95%*CI:* 1.29−1.65), and all-cause mortality (*RR:* 1.50, 95%*CI:* 1.29−1.74). In the continuous analysis, per doubling of FGF23 was associated with increased risks of MI (*RR:* 1.08, 95%CI: 0.94−1.25), stroke (*RR:* 1.21, 95%*CI*: 0.99−1.48), HF (*RR:* 1.24, 95%*CI*: 1.14−1.35), CVD events (*RR:* 1.12, 95%*CI:* 0.99−1.27), cardiovascular mortality (*RR:* 1.43, 95%*CI:* 1.09−1.88), all-cause mortality (*RR:* 1.37, 95%*CI:* 1.15−1.62). Furthermore, the dose-response analysis demonstrated a potentially non-linear relationship between FGF23 and stroke, HF, and all-cause mortality. In contrast, a potentially linear relationship between FGF23 and cardiovascular mortality was observed (*p for non-linearity* = 0.73).

**Conclusion:**

The present study suggests that increased serum FGF23 levels are positively related to CVD events and mortality in the general population. The clinical application of FGF23 levels to predict CVD risk requires further research.

## Introduction

Cardiovascular diseases (CVDs) remain the leading cause of mortality worldwide, resulting in 17.3 million deaths each year. The number of annual deaths is expected to exceed 23.6 million by 2030. Meanwhile, cardiovascular diseases are responsible for approximately 40% of deaths in the Chinese population (1, 2). Therefore, it is essential to explore the prevention and treatment of CVDs and develop effective solutions.

Fibroblast growth factor 23 (FGF23) is a phosphaturic hormone primarily secreted by osteocytes and osteoblasts. It participates in adjusting systemic phosphate homeostasis, vitamin D metabolism, and a-Klotho expression through the bone-kidney axis (3, 4). FGF23 mainly exerts physiological effects in the kidneys and the parathyroid gland by binding to the FGF receptor (FGFR) and its co-receptor klotho (5). The main physiological role of FGF23 is to enhance urinary phosphate excretion, decrease the 1,25-dihydroxy Vitamin D levels *in vivo*, and suppress the secretion of parathyroid hormone (PTH) (6, 7). In the past decade, FGF23 has been recognized as an important biomarker of cardiovascular diseases. Furthermore, CVD is also the leading cause of death in patients with chronic kidney disease (CKD). The serum FGF23 levels in CKD patients were significantly higher than in healthy populations and demonstrated an increase with decreasing glomerular filtration rate (GFR) (8, 9). Previous meta-analyses have investigated the relationship between FGF23 and CVDs in CKD patients (10–13), but the association and dose-response in the general population remain unclear. Therefore, this systematic review and dose-response meta-analysis explored of the association between FGF23 levels and cardiovascular diseases and mortality risk in the general population.

## Methods

This review strictly followed the Preferred Reporting Items for Systematic reviews and Meta-Analysis (PRISMA) and G-Dose checklists guidelines (Supplementary Tables 1, 2). The study was registered in PROSPERO (CRD42021281837).

### Literature search

Two authors (PX and ML) performed a literature search using the PubMed, EMBASE, and the Cochrane Library databases, including articles published before 10 September 2022. Medical subject headings were combined with free-text terms for retrieval without language restrictions. The search conditions were as follows: “fibroblast growth-factor 23 OR FGF23 protein OR fibroblast growth factor 23 OR FGF23 protein OR phosphatonin OR tumor-derived hypophosphatemia inducing factor” And “cardiovascular diseases OR cardiovascular disease OR disease, cardiovascular OR diseases, cardiovascular OR myocardial infarction OR stroke OR heart failure OR atrial fibrillation OR coronary heart disease OR left ventricular hypertrophy OR hypertension.” The details of the search strategy are described in Supplementary Table 3.

### Study selection

According to the PICOS (population, intervention, comparison, outcome, and study design) strategy, the inclusion criteria for this review were as follows:

(1) The participants were adults from the general population (age > 18 years).

(2) The studies compared high vs. low FGF23 levels.

(3) The outcomes included all kinds of cardiovascular diseases (including myocardial infarction, stroke, heart failure, atrial fibrillation, coronary heart disease, left ventricular hypertrophy, hypertension, composite of cardiovascular events, cardiovascular mortality, and all-cause mortality).

(4) Prospective cohort studies were included.

Adjusted relative risk (*RR*) or hazard ratio (*HR*), and the corresponding 95% confidence interval (*CI*) were required. Prospective case-cohorts were regarded as prospective cohort studies (14). Case-control or cross-sectional study, reviews, case reports, abstracts, letters or comments, and animal research were excluded. The Newcastle-Ottawa Scale (NOS) was used to assess the quality of the included studies (15), and studies with moderate-to-high risk of bias were included (score > 6). The reasons for study exclusion are detailed in Supplementary Table 4.

### Data extraction

Two authors (PX and ML) independently recorded the following information for each related study: first author, country, publication year, study design, sample size, sex, mean or median age, follow-up duration, FGF23 categories, outcomes reported, FGF23 measurement, *RR* or *HR* with the 95%*CI*, and adjusted co-founders.

### Statistical analysis

In prospective studies, *HR* was considered to be equivalent to *RR*. The adjusted *RR* was transformed to the natural logarithms (log*RR*) to fit a normal distribution, and the standard errors (SElog [*RR*]) were calculated according to the corresponding 95%*CI*s. The random effects model was applied to pool the risk estimates considering the heterogeneity of different cohort studies. When an included study compared the lowest level or the reference category of FGF23 with the higher categories (≥2 categories), the highest category was regarded as the high level, while the reference level or the lowest level was regarded as the low level. For the category analysis, the summary *RR*s and 95%*CI*s were calculated by comparing the highest level of FGF23 to the lowest level of FGF23. For the dose-response analysis, the method described by Greenland and Longnecker (16) was used, with linear trends per 20 RU/mL increment of FGF23. The study-specific slopes and 95%*CI*s for FGF23 were calculated from the natural logs of the *RR*s and *CI*s. The FGF23 results were converted into RU/mL for all the included studies (1 RU/mL is approximately equivalent to 2 pg/mL) (17). FGF23 was also unified as a continuous variable into log base 2 transformations, interpreted as “per doubling” to calculate the corresponding summary *RR*s and 95%*CI*s. For the non-linear analysis, the robust error meta-regression method (REMR) developed by Xu and Doi (18, 19) was applied. The method requires data on the levels of FGF23 doses and RRs with variance estimates for at least two quantitative dose categories. If the levels of FGF23 doses was not directly reported, the mean or median of each FGF23 level between the upper and lower boundaries in each category was used to estimate the corresponding dose for each study. For open terminal categories, the open interval was set to the same length as that of the adjacent interval (20, 21).

The presence of heterogeneity between studies was estimated using the Cochrane *Q* test and the *I*^2^ statistic. For the *Q* statistic, *P* < 0.1 indicated significant heterogeneity. For the *I*^2^ statistic, <25% indicated low or no heterogeneity; 25%−50% suggested moderate heterogeneity; >50% was considered high heterogeneity (22). For those outcomes which a number of included studies over 6, pre-defined subgroups were stratified by age (≤60 years vs. >60 years), follow-up duration (≤10 years vs. >10 years), FGF23 measurement (iFGF23 vs. cFGF23). All statistical analyses were performed using Review Manager (RevMan) version 5.3 (The Cochrane Collaboration 2014; Nordic Cochrane Center Copenhagen, Denmark) and STATA (Version 16.0, Stata Corp., LP, College Station, TX, United States) software. All *P*-values were two-sided, and *P*-value < 0.05 was considered statistically significant.

## Results

### Study search and selection

**FIGURE 1:**
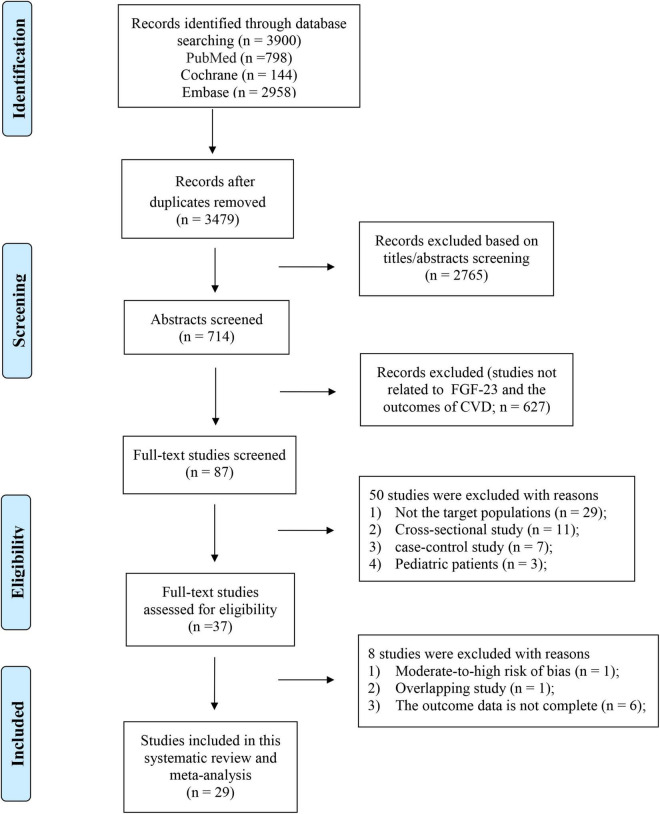
Flowchart of the study selection investigating the association between levels of FGF23 and risk of CVDs in the general population.

We initially identified 3900 (PubMed = 798, EMBASE = 2958, Cochrane Library = 144) articles from the electronic literature search. After removing irrelevant and duplicate articles, a full-text review was performed for the remaining 87 potentially relevant studies. Ultimately, 29 articles were identified for the meta-analysis. The reasons for exclusion (*n* = 58) are detailed in Supplementary Table 3 and the details of the study selection are listed in Figure 1.

### Study characteristics and quality

The baseline characteristics of the included studies are summarized in Table 1. Among the included studies, 23 studies reported associations between FGF23 and CVD risk in the general population: [myocardial infarction (MI) = 4, stroke = 6, heart failure (HF) = 10, CVD events = 6, sudden cardiac death (SCD) and Non-SCD = 1, atrial fibrillation (AF) = 2, hypertension = 3, coronary heart disease (CHD) = 3, left ventricular hypertrophy (LVH) = 1]. Furthermore, 11 studies investigated the association between FGF23 and all-cause mortality, and 8 studies assessed the association between FGF23 and cardiovascular mortality. In total, 18 studies presented the C-terminal FGF23 levels, and 14 studies reported intact-FGF23 levels. The sample sizes ranged from 727 to 22,127, with a total of 135,576 participants included. The duration of follow-up ranged from 1.75 years to 18.6 years. The included studies were of moderate-high quality, with NOS scores of 6 or higher (Supplementary Table 5).

**TABLE 1:** Basic characteristics of the articles included in this systematic review and meta-analysis of FGF23 and the risk of cardiovascular diseases and mortality in the general population.

Author, year, Country	Study acronym	Study design	Number of patients	Follow-up time	Baseline demographics	Type of FGF-23	Outcomes reported	RR/OR(95%CI); comparison	Measurement of FGF23	Adjustment for covariates
Di Giuseppe, 2015, Germany (23)	EPIC-Germany	Prospective Case-cohort	2908	Mean: 8.2 yr	Age: 52	C-terminal	Myocardial infarction	1.62(1.07-2.45);Q4vsQ1	ELISA	age, sex, laboratory batch, smoking, eGFR, PTH, 25(OH)D3, dietary
					Men:50%			1.27(1.05-1.53);log/unit	(immutopics)	calcium, phosphorus intake, prevalent hypertension,
							Stroke	1.3(0.95-1.86);Q4vsQ1		hyperlipidemia,
								1.13(0.96-1.31);log/unit		diabetes.
							CVD events	1.36(1.02-1.82);Q4vsQ1		
								1.16(1.02-1.32);log/unit		
Ärnlöv, 2013,Uppsala, Sweden (36)	PIVUS	Prospective cohort	1003	Median: 5.1 yr	Age: 70 yr	Intact	CVD events	1.68(0.97-2.9);Q4vsQ1	ELISA	age, sex, eGFR, PTH, vitaminD, calcium, phosphate, markers of
					Men:50%				(kainos)	cardiovascular pathology.
Ärnlöv, 2012, Uppsala, Sweden (46)	ULSAM	Prospective cohort	727	Median: 9.7 year	Age: 78 yr	Intact	All-cause mortality	1.37(0.85-2.16);Q5vsQ1	ELISA	Age, established cardiovascular risk factors, GFR, secondary models, LVH,
					Men:100%		Cardiovascular mortality	1.56(0.76-3.18);Q5vsQ1	(kainos)	albuminuria, markers of inflammation, antioxidative stress.
Brandenburg, 2014, Germany (47)	LURIC	Prospective cohort	2974	Median: 9.9 year	Age: 63 yr	C-terminal	All-cause mortality	1.72(1.38-2.14);Q4vsQ1	ELISA (immutopics)	Age, gender, coronary artery disease, BMI, Type 2 diabetes, hypertension, smoking
					Men:69%		Cardiovascular mortality	1.57(1.19-2.07);Q4vsQ1		status, GFR, use of lipidlowering drugs, LDL-C, HDL-C,
								1.35(1.2-1.52); FGF23/SD(28.18RU/ml)		triglycerides, phosphate, alkaline phosphatase, calcium, PTH, 25-hydroxyvitamin D.
Di Giuseppe, 2014, Germany (32)	EPIC-Potsdam	Prospective Case-cohort	1443	Mean: 8 yr	Age: 52	C-terminal	Heart failure	1.89(1.03-3.46);Q5vsQ1	ELISA (immutopics)	sex, fasting status, waist circumference, BMI, alcohol
					Men:44%			1.29(1.07-1.56);log/unit		consumption, sports activity, smoking status, educational level, prevalent hypertension, diabetes, hyperlipidemia, CHD, PTH, 25(OH)D3, eGFR, stratified by baseline age.
Ix, 2012, USA (24)	CHS	Prospective cohort	3107	Median: 10.5 yr	Age: 78 yr	C-terminal	Myocardial infarction	1.19(0.77-1.83);Q4vsQ1	ELISA (immutopics)	age, sex, race, health status, current smoking, prior stroke,
					Men:40%			0.97(0.8-1.18);log/unit		prior MI, prior HF, prior
							Stroke	1.05(0.68-1.61);Q4vsQ1		claudication, hypertension,
								0.99(0.81-1.2);log/unit		diabetes, BMI, estrogen use
							Heart failure	1.37(0.99-1.89);Q4vsQ1		(women), total chol, lipid med use, natural log (CRP).
								1.17(1.02-1.33);log/unit		
							CVD events	1.07(0.79-1.45);Q4vsQ1		
								0.99(0.87-1.13);log/unit		
							All-cause mortality	1.29(1.05-1.59);Q4vsQ1		
								1.07(0.98-1.17);log/unit		
							Cardiovascular mortality	1.28(0.89-1.84);Q4vsQ1		
								1.05(0.89-1.23);log/unit		
Kestenbaum, 2014, USA (27)	MESA	Prospective cohort	6547	Median: 8.5 yr	Age: 62 yr	Intact	Stroke	1(0.61-1.65);Q4vsQ1	ELISA	age, sex, study site, height, weight, diabetes, SBP, any hypertension
					Men:47%		Heart failure	1.72(1.06-2.8);Q4vsQ1	(kainos)	medication, current smoking
								1.19(1.03-1.37);FGF23/per 20pg/ml increase		C-reactive protein concentration, education level, eGFR, log(urine
							Coronary heart disease	1.39(1-1.92);Q4vsQ1		albumin to creatinine ratio).
Lutsey, 2014, USA (33)	ARIC	Prospective cohort	11638	Median: 18.6 yr	Age: 57 yr	Intact	Heart failure	1.3(1.13-1.51);Q5vsQ1	ELISA	age, sex, race, education, physical activity, smoking, BMI, prevalent
					Men:43%			1.08(1.04-1.13); FGF23/SD(16.4pg/ml)	(kainos)	diabetes, SBP, HTN medication use, lipid medication use, LDL cholesterol, HDL
							Cardiovascular mortality	1.28(1.04-1.57);Q5vsQ1		cholesterol, eGFR category.
								1.08(1.02-1.15);FGF23/SD(16.4pg/ml)		
							Coronary heart disease	1.32(1.11-1.56);Q4vsQ1		
Panwar, 2015, USA (28)	REGARDS	Prospective Case-cohort	1551	7yr	Age: 65 yr	C-terminal	Stroke	1.19(0.78-1.82);Q4vsQ1	ELISA (immutopics)	age, race, age x race interaction, sex, SBP, diabetes, cigarette
					Men:45%					smoking, coronary heart disease, AF, LVH, plasma phosphorus, plasma calcium,
										eGFR, natural log-transformed albumin to creatinine ratio.
Parker, 2010, San-Francisco, USA (25)	HSS	Prospective cohort	833	Median: 6.0 year	Age: 67 yr	C-terminal	Myocardial infarction	1.05(0.85-1.3);ln/unit	ELISA (immutopics)	age, sex, race, waist-to-hip ratio, smoking, hypertension, diabetes,
					Men:81%		Stroke	1.5(1.11-2.04);ln/unit		SBP, DBP, eGFR, total and
							Heart failure	1.31(1.08-1.59);ln/unit		high-density lipoprotein
							CVD events	1.83(1.14-2.94);T3vsT1		cholesterol levels, C-reactive
								1.24(1.06-1.44);ln/unit		protein level, ejection fraction,
							All-cause mortality	2.07(1.36-3.13);T3vsT1		peak exercise capacity, use of
								1.4(1.22-1.6);ln/unit		medicine, calcium level, phosphorus level, ucMGP, fetuin-A.
Westerberg, 2013, Sweden (50)	MrOS	Prospective cohort	2838	Mean: 4.5 yr	Age: 75.5 yr	Intact	All-cause mortality	1.13(0.8-1.59);Q4vsQ1	ELISA	age, BMI, eGFR, albumin, calcium, phosphate, (PTH,
					Men:100%			1.02(0.89-1.17);log/SD (0.19log pg/ml)	(kainos)	FGF23 or 25D as appropriate), smoking, diabetes, hypertension,
							Cardiovascular mortality	1.37(0.74-2.51);Q4vsQ1		prevalent cancer, prevalent CVD.
								1.26(0.99-1.59);log/SD(0.19log pg/ml)		
Wright, 2014, USA (29)	NOMAS	Prospective cohort	2525	Mean: 12 yr	Age: 69 yr	C-terminal	Stroke	1.4(1-1.9);H2vsH1	ELISA (immutopics)	age, sex, race/ethnicity, eGFR, SBP, DBP, medication use, fasting
					Men:36%			1.3(1.1-1.6);ln/unit		total cholesterol, tobacco use, moderate alcohol use, BMI, phosphate, parathyroid hormone.
Souma, 2016, USA (49)	NOMAS	Prospective cohort	2525	Mean: 14 yr	Age: 69 yr	C-terminal	All-cause mortality	2.71(1.3-5.65);ln/unit	ELISA (immutopics)	age, sex, race/ethnicity, traditional cardiovascular disease
					Men:36%		Cardiovascular mortality	2.07(1.45-2.94);Q5vsQ1		risk factors (cigarette smoking, BMI, hypertension, diabetes,
								1.38(1.19-1.6);ln/unit		hypercholesterolemia, prevalent cardiovascular disease), eGFR, mineral metabolism markers (phosphate, PTH,
										25-hydroxyvitamin D, calcium, albumin).
Almahmoud, 2018, USA (31)	MESA	Prospective cohort	6542	Median: 12.1 yr	Age: 62 yr	Intact	Heart failure	1.51(1-2.3);Q4vsQ1	ELISA	age, sex, race/ethnicity, education, study site, height, weight, SBP,
					Men:47%			1.18(1.02-1.37);FGF23/per 20pg/ml increase	(kainos)	antihypertensive medications, DM, smoking, C-reactive protein, UACR, eGFR, NT-proBNP, 25(OH) vitamin D, PTH, phosphate.
De Jong, 2021, Netherlands (51)	PREVEND	Prospective cohort	5253	Median: 8.4 yr	Age:52yr	C-terminal	All-cause mortality	1.99(1.33-2.98);T3vsT1	ELISA	age, sex, mean arterial blood pressure, the use of
					Men:47%			1.3(1.03-1.63);log/unit	(Quidel)	antihypertensive drugs, BMI, ethnicity, smoking status, eGFR, albuminuria, total cholesterol, history of diabetes, serum phosphate, calcium, plasma PTH, 25(OH)D, hsCRP, 24-h urinary urea excretion, serum iron, transferrin, ferritin.
Sharma, 2021, USA (48)	HABC	Prospective cohort	2763	Median:8.3 yr	Age:75yr	Intact	All-cause mortality	1.31(1.05-1.62);Q4vsQ1	ELISA	age, gender, race, site, education, diabetes, SBP, HTNmeds, BMI,
					Men:45%			1.24(1.12-1.37);log/unit	(kainos)	smoking, prevalent CVD,
							Cardiovascular mortality	1.54(1.08-2.18);Q4vsQ1		albumin, CRP, statin use, total cholesterol, calcium, phosphate,
								1.31(1.11-1.54);log/unit		PTH, eGFR, UACR.
Robinson-Cohen, 2020, USA (34)	MESA	Prospective cohort	6413	Median:14.9yr	Age:62yr	Intact	Heart failure	1.37(1.07-1.75);H2vsH1	ELISA	age, gender, gross family income, educational attainment,
					Men:47%				(kainos)	race/ethnicity, BMI, SBP, use of medication, low-density lipoprotein, total cholesterol, diabetes status, smoking status, eGFR.
Binnenmars, 2022, Netherlands (35)	PREVEND	Prospective cohort	6830	Median: 7.4yr	Age:54yr	C-terminal	Heart failure	1.36(0.93-2.00);T3vsT1	ELISA	age, sex, White race, BMI, smoking, alcohol use,
					Men:49.7%			1.29(1.06-1.57);log/unit	(Quidel)	hypercholesterolemia, hypertension, diabetes type 2, myocardial infarction, atrial
										fibrillation, eGFR, urinary albumin excretion, high- sensitivity CRP, hemoglobin, ferritin, transferrin saturation, NT-proBNP.
Paul, 2021, USA (30)	CARDIA	Prospective cohort	3151	Median: 7.6yr	Age:45yr	C-terminal & Intact	CVD events	0.99(0.61-1.6);Q4vsQ1 &	ELISA	age, sex, race, educational attainment, smoking status,
					Men:44%			0.71(0.45-1.13);Q4vsQ1	(Quidel)	physical activity, BMI, diabetes
								1.14(0.97-1.34);log/unit &		mellitus, SBP, antihypertensive
								0.82(0.62-1.08);log/unit		drug use, total cholesterol,
							Stroke	1.15(0.5-2.62);Q4vsQ1 &		HDL-C, statin use, eGFR, UACR.
								0.47(0.21-1.02);Q4vsQ1		
								1.18(0.95-1.47);log/unit &		
								0.58(0.34-0.97);log/unit		
							Heart failure	2.66(0.89-7.95);Q4vsQ1 &		
								0.74(0.27-2.04);Q4vsQ1		
								1.52(1.18-1.96);log/unit &		
								0.86(0.5-1.49);log/unit		
							All-cause mortality	1.27(0.79-2.06);Q4vsQ1 &		
								0.55(0.33-0.92);Q4vsQ1		
								1.17(1-1.38);log/unit &		
								0.86(0.64-1.17);log/unit		
Haring, 2016, USA (37)	FHS	Prospective cohort	3236	Median:10.8yr	Age:59yr	C-terminal	CVD events	1.17(0.87-1.59);Q4vsQ1	ELISA (immutopics)	age, sex, BMI, SBP, antihypertensive medication,
					Men:46%			1.05(0.94-1.17);ln/SD (0.33logRU/ml)		total and high-density lipoprotein cholesterol ratio, smoking, type 2
							All-cause mortality	1.87(1.38-2.53);Q4vsQ1		diabetes mellitus, cohort.
								1.31(1.2-1.42);ln/SD (0.33logRU/ml)		
							Cardiovascular mortality	1.41(0.78-2.55);Q4vsQ1		
								1.32(1.09-1.59);ln/SD (0.33logRU/ml)		
Sharma, 2020, USA (26)	CHS	Prospective Case-cohort	844	10yr	Age:78yr	Intact &	Myocardial infarction	0.99(0.75-1.31);log/unit	ELISA	ferritin, Transferrin Saturation, CRP, UACR, eGFR
					Men:38%	C-terminal	Heart failure	1.19(0.95-1.50);log/unit	(kainos) &	
							All-cause mortality	1.11(0.97-1.28);log/unit	ELISA (immutopics)	
Deo, 2015, USA (45)	CHS	Prospective cohort	3244	Median:8.1yr	Age:78yr	C-terminal	Sudden cardiac death	1.01(0.69-1.48);log/unit	ELISA (immutopics)	age, sex, race, diabetes, hypertension, congestive heart
					Men:40%		Non-sudden cardiac death	1.02(0.85-1.22);log/unit		failure, myocardial infarction, smoking, and alcohol use, eGFR, natural log(ACR).
Mathew, 2014, USA (38)	MESA & CHS	Prospective cohort	6398 & 1350	Median:7.7yr & 8yr	Age:62yr	Intact &	Atrial fibrillation	1.38(0.94- 2.04);Q4vsQ1	ELISA	age, gender, race/ethnicity, study site, attained education, low
					Men:46.5%	C-terminal		&	(kainos) &	density cholesterol, use of
					&			1.52(1-2.32);Q4vsQ1	ELISA (immutopics)	lipid-lowering medications, current smoking, diabetes,
					Age:77yr					physical activity, height, height
					Men: 29%					squared, weight, UACR, eGFR, SBP, and use of hypertension medication, the serum concentrations of calcium, phosphate, 25-hydroxyvitamin D, PTH.
Alonso, 2014, USA (39)	ARIC	Prospective cohort	12349	Mean:17yr	Age:57yr	Intact	Atrial fibrillation	1.1(0.95-1.27);Q4vsQ1	ELISA	age, race, sex, study site, BMI, smoking, education, height,
					Men:43%				(kainos)	diabetes, SBP, DBP, use of antihypertensive medication, prevalent coronary heart disease, prevalent heart failure, ECG-based left ventricular hypertrophy, NT-proBNP, high-sensitivity C-reactive protein, eGFR, serum calcium, phosphorus, PTH, 25-hydroxyvitamin D.
Akhabue, 2018, USA (40)	CARDIA	Prospective cohort	1758	5yr	Age:62yr	C-terminal	Hypertension	1.45(1.18- 1.77);Q4vsQ1	ELISA (immutopics)	age, sex, race, education, study center, BMI, smoking status,
					Men:46.5%					physical activity, Triglyceride/high density lipoprotein ratio, eGFR, UACR.
Fyfe-Johnson, 2016, USA (41)	ARIC	Prospective cohort	7948	Median: 5.9yr	Age:62yr	Intact	Hypertension	1.21(1.08- 1.35);D10vsQ1	ELISA	age, race, sex, ARIC field center, educational attainment, cigarette
					Men:46.5%				(kainos)	smoking, alcohol intake,
										physical activity, BMI, serum phosphorus, eGFR category.
Drew,2020, USA (42)	HABC	Prospective cohort	2496	10yr	Age:75yr	Intact	Hypertension	1.69(1.31- 2.18);Q4vsQ1	ELISA	age, sex, race, diabetes, cardiovascular disease, eGFR,
					Men:49%				(kainos)	UACR, BMI, smoking, calcium, phosphorus, 25(OH) Vitamin D, PTH.
panwar, 2018, USA (43)	REGARDS	Prospective Case-cohort	22127	4yr	Age:64yr	C-terminal	Coronary heart disease	2.15(1.35-3.42);Q4vsQ1	ELISA (immutopics)	race, age, sex, BMI, SBP level, DBP level, diabetes, physical
					Men:41%					activity, income, education, neighborhood socioeconomic characteristics, cigarette smoking,
										LVH, use of medications, eGFR, natural log–transformed albumin to creatinine ratio, natural log–transformed CRP level, intact parathyroid hormone concentration, triglyceride level, high-density lipoprotein cholesterol level, total cholesterol level.
Jovanovich, 2013, USA (44)	CHS	Prospective cohort	2255	3yr	Age:62yr	C-terminal	Left ventricular hypertrophy	1.5(0.91- 2.64);Q4vsQ1	ELISA (immutopics)	age, sex, race, clinic site, weight, smoking status, diabetes, use of
					Men:46.5%					antihypertensive medications, SBP, CRP.

NR, not reported; ELISA, enzyme linked immunosorbent assay; BMI, body mass index; BP, blood pressure; CRP, C-reactive protein; UACR, urine albumin creatine ratio; HbA1c, Glycosylated Hemoglobin; LDL-C, low-density lipoprotein cholesterol; HDL-C, high-density lipoprotein cholesterol; NT-proBNP, N-terminal of the prohormone B-type natriuretic peptide; eGFR, estimated glomerular filtration rate; PTH, parathyroid hormone; GDF-15, growth differentiation factor 15; EPIC, European Prospective Investigation into Cancer and Nutrition; AF, atrial fibrillation; COPD, chronic obstructive pulmonary disease; CVD, cardiovascular diseases; DM, diabetes mellitus; EPIC, European Prospective Investigation into Cancer and Nutrition (EPIC); PIVUS, Prospective Investigation of the Vasculature in Uppsala Seniors study; ULSAM, Uppsala Longitudinal Study of Adult Men; CHS, Cardiovascular Health Study; MESA, Multi-Ethnic Study of Atherosclerosis; ARIC, Atherosclerosis Risk in Communities Study; REGARDS, Reasons for Geographic and Racial Differences in Stroke; HSS, Heart and Soul Study; FHS, Framingham Heart Study; NOMAS, Stroke-free North Manhattan Study; MrOS, multicenter prospective Osteoporotic Fractures in Men study; CRIC, Chronic Renal Insufficiency Cohort; HOST, Homocysteine in Kidney and End Stage Renal Disease study; HEMO, Hemodialysis Study; EVOLVE, Evaluation of Cinacalcet Hydrochloride Therapy to Lower Cardiovascular Events; LURIC, Ludwigshafen Risk and Cardiovascular Health study; PREVEND, Prevention of Renal and Vascular Endstage Disease study; CARDIA, Coronary Artery Risk Development in Young Adults Study; HABC, Health, Aging, and Body Composition Study.

### Association between fibroblast growth factor-23 and risk of cardiovascular diseases

**FIGURE 2:**
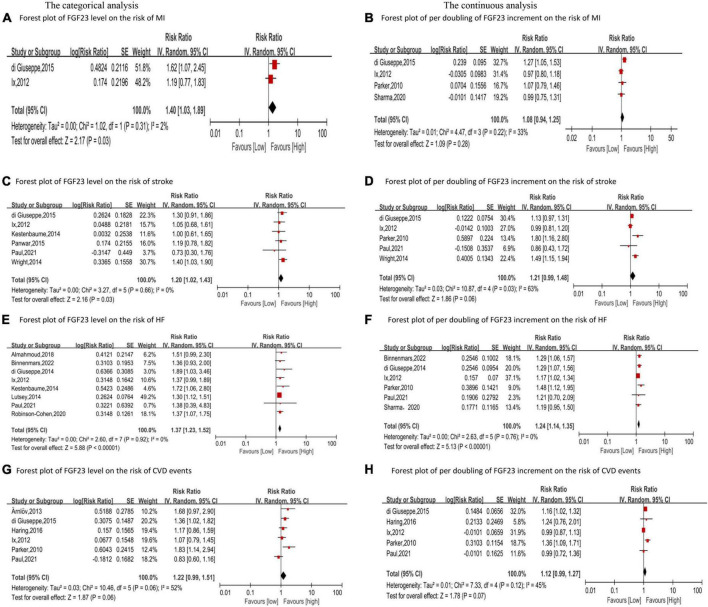
Forest plot for the association between FGF23 level and the risk of MI **(A)**, stroke **(C)**, HF **(E)**, and CVD events **(G)** in the general population, analyzed as category variable, highest vs. lowest; the association between per doubling of FGF23 increment and the risk of MI **(B)**, stroke **(D)**, HF **(F)**, and CVD events **(H)** in the general population, analyzed as a continuous variable.

Four studies (23–26) reported the relationship between FGF23 and MI in the general population. The categorical analysis revealed that high FGF23 levels were related to increased risk of MI (*RR*: 1.40, 95%*CI*:1.03−1.89, *p* = 0.03; Figure 2A), with low heterogeneity (*p* = 0.31, *I*^2^ = 2%). Four studies reported continuous analysis (23–26); the *RR* of MI per doubling of FGF23 was 1.08 (95%*CI*: 0.94−1.25, *p* = 0.28; Figure 2B), with moderate heterogeneity (*p* = 0.22, *I*^2^ = 33%).

**FIGURE 3:**
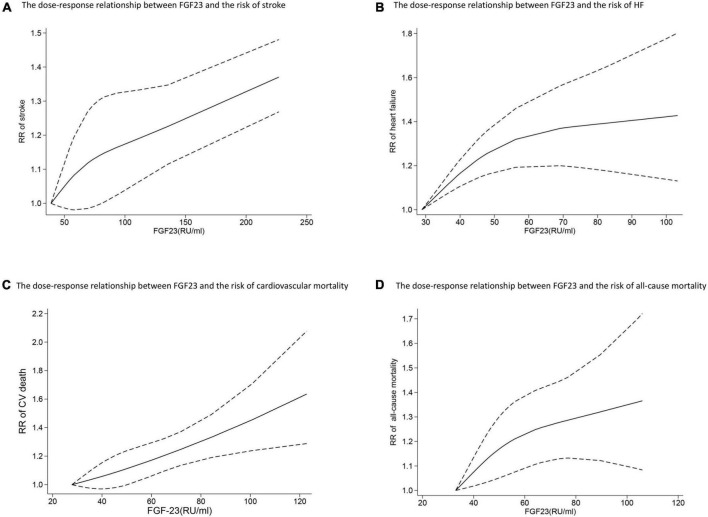
The dose-response relationship between FGF23 levels and the risk of stroke **(A)**, HF **(B)**, cardiovascular mortality **(C)**, and all-cause mortality **(D)** in the general population. FGF23 levels were converted to RU/ml and the results were pooled in a one-stage random-effects model. The bold lines indicate the pooled restricted cubic spline model and the black dashed line indicates the 95% CIs of the pooled curve.

Six studies analyzed the relationship between FGF23 levels and stroke (23, 24, 27–30). High FGF23 levels were related to increased risk of stroke in the categorical analysis (*RR*: 1.20, 95%*CI*: 1.02−1.43, *p* = 0.03; Figure 2C), without heterogeneity (*p* = 0.66, *I*^2^ = 0%). In the continuous analysis, the *RR* of stroke per doubling of FGF23 was 1.21 (95%*CI*: 0.99−1.48, *p* = 0.06; Figure 2D), with high heterogeneity (*p* = 0.02, *I*^2^ = 63%). Moreover, six studies performed a dose-response analysis (23, 24, 27–30), revealing a non-linear association between FGF23 and stroke (*p for non-linearity* = 0.10; Figure 3A).

Ten studies (24–27, 30–35) reported the association between FGF23 levels and HF in general populations. A significant increase in HF risk was associated with high FGF23 levels (*RR*: 1.37, 95%*CI*: 1.23−1.52, *p* < 0.00001; Figure 2E), without heterogeneity (*p* = 0.92, *I*^2^ = 0%). In the continuous analysis, the summary *RR* for a 20 RU/ml increment of FGF23 was 1.25 (95%*CI*: 1.14−1.37, *p* < 0.00001; Supplementary Figure 1A), without heterogeneity (*p* = 0.47, *I*^2^ = 0%); and the *RR* of HF per doubling of FGF23 was 1.24 (95%*CI*: 1.14−1.35, *p* < 0.00001; Figure 2F), without heterogeneity (*p* = 0.76, *I*^2^ = 0%). In addition, eight studies carried out a dose-response analysis (24, 27, 30–35), and a potentially non-linear association of FGF23 was observed with HF (*p* for non-linearity = 0.001; Figure 3B).

Additionally, six studies (23–25, 30, 36, 37) considered the composite of MI, stroke, heart failure, and so on as CVD events, assessing FGF23 levels and CVD events in general populations. High FGF23 levels were related to an increased risk of CVD events (*RR*: 1.22, 95%*CI*: 0.99−1.51, *p* = 0.06; Figure 2G), with high heterogeneity (*p* = 0.06, *I*^2^ = 52%). In the continuous analysis, the *RR* of CVD per doubling of FGF23 was 1.12 (95%*CI*: 0.99−1.27, *p* = 0.07; Figure 2H), with moderate heterogeneity (*p* = 0.12, *I*^2^ = 45%).

A few studies examined the relationship between FGF23 and other cardiovascular diseases, but the results were not pooled due to the scarcity of data. As shown in Table 1, two studies (38, 39) reported the relationship between FGF23 levels and atrial fibrillation, including 2,092 cases out of 20,097 participants. Mathew et al. (38) described the association between FGF23 and AF incidence in The Multi-Ethnic Study of Atherosclerosis (MESA) and the Cardiovascular Health Study (CHS) including 291 MESA patients (HR [quartile 4 vs. quartile 1]: 1.38) and 229 CHS patients (HR [quartile 4 vs. quartile 1]: 1.52) adjusted for potential confounding characteristics. However, Alonso et al. (39) revealed that baseline FGF23 levels were not associated with AF risk, regardless of kidney function. This study summarized data from 1,572 patients (HR [quartile 4 vs. quartile 1]:1.1) adjusted for potential confounding factors. Moreover, three studies (40–42) investigated the relationship between FGF23 levels and hypertension. The study from Akhabue et al. (40) included 618 patients and showed that elevated FGF23 levels were related to an increased risk of hypertension in fully adjusted models (RR [quartile 4 vs. quartile 1]: 1.45). In another cohort study, Fyfe-Johnson et al. (41) demonstrated that the HR for hypertension was 1.21 for decile 10 compared to quintile 1 after adjusting for demographics, behaviors, and adiposity. Drew et al. (42) reported that FGF23 was related to an increased hypertension risk after adjustments, including 576 patients (RR [quartile 4 vs. quartile 1]: 1.69).

Three studies (27, 33, 43) reported the relationship between FGF23 levels and the risk of CHD, including 2,317 cases. Panwar et al. (28) evaluated 829 patients adjusted for established CHD risk factors and kidney function, suggesting that elevated FGF23 concentrations were related to an increased CHD risk (HR [quartile 4 vs. quartile 1]: 2.15). In addition, Kestenbaum et al. (27) revealed that elevated FGF23 concentrations were related to an increased CHD risk, including 363 patients (RR [quartile 4 vs. quartile 1]: 1.39) after adjustments. Another cohort study involving 1125 patients with CHD reported similar results after adjustments (*RR:* 1.32).

Jovanovich et al. (44) found that FGF23 was associated with greater risk of LVH, including 310 patients (OR [quartile 4 vs. quartile 1]: 1.5) in adjusted analyses. Deo et al. (45) investigated 570 cases among the elderly population and observed that FGF23 elevations were independently associated with non-SCD (HR [quartile 4 vs. quartile 1]: 1.02) after adjustments.

### Association between fibroblast growth factor-23 and death

**FIGURE 4:**
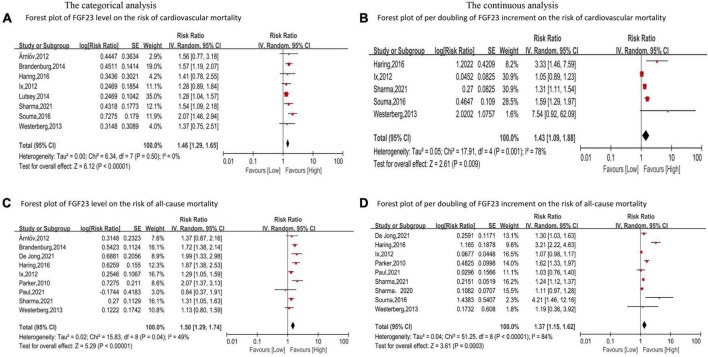
Forest plot for the association between FGF23 levels and the risk of cardiovascular mortality **(A)** and all-cause mortality **(C)** in the general population, analyzed as category variable, highest vs. lowest; the association between per doubling of FGF23 increment and the risk of cardiovascular mortality **(B)**, and all-cause mortality **(D)** in the general population, analyzed as a continuous variable.

Eight studies (24, 33, 37, 46–50) reported the association between FGF23 and cardiovascular mortality. The categorical analysis indicated that high FGF23 concentrations were related to increased risk of cardiovascular mortality (*RR*: 1.46, 95%*CI*: 1.29−1.65, *p* < 0.00001; Figure 4A), without heterogeneity (*p* = 0.50, *I*^2^ = 0%). In the continuous analysis, the summarized *RR* for a 20 RU/ml increment of FGF23 was 1.23 (95%*CI*: 1.15−1.32, *p* < 0.00001; Supplementary Figure 1B), without heterogeneity (*p* = 0.83, *I*^2^ = 0%); and the *RR* of cardiovascular mortality per doubling of FGF23 was 1.43 (95%CI: 1.09−1.88, *p* = 0.009; Figure 4B), with high heterogeneity (*p* = 0.001, *I*^2^ = 78%). Moreover, eight studies (24, 33, 37, 46–50) were included in the dose-response analysis, revealing a significant linear dose-response relationship between FGF23 levels and cardiovascular mortality (*p for non-linearity* = 0.73; Figure 3C).

In addition, eleven studies (24–26, 30, 37, 46–51) evaluated the association between FGF23 and all-cause mortality in general populations. A significant increase in risk of death was associated with high FGF23 levels (*RR*: 1.50, 95%*CI*: 1.29−1.74, *p* < 0.00001; Figure 4C), with moderate heterogeneity (*p* = 0.04, *I*^2^ = 49%). The *RR* of all-cause mortality per doubling of FGF23 was 1.42 (95%*CI*: 1.37−1.15, *p* = 0.0003; Figure 4D), with high heterogeneity (*p* < 0.00001, *I*^2^ = 84%). Furthermore, nine studies were included in the dose-response analysis (24, 25, 30, 37, 46–48, 50, 51), and a non-linear association of FGF23 with all-cause mortality was observed (*p for non-linearity* = 0.10; Figure 3D).

Moreover, the above results were confirmed by the subgroup analysis based on age (≤60 years *vs.* > 60 years), follow-up duration (≤10 years *vs.* > 10 years), and FGF23 measurement (iFGF23 *vs.* cGFG23) (Supplementary Figures 2, 3).

### Sensitivity analysis and publication bias

Deleting individual studies in the sensitivity analysis did not significantly alter the pooled effect size (Supplementary Figure 4). The absence of publication bias was presented by using Egger’s test (*p* = 0.394 and 0.530) and a Funnel plot (Supplementary Figure 5).

## Discussion

The present study showed a significant relationship between FGF23 levels and the risk and mortality of CVDs in the general population. The dose-response analysis suggested a potentially non-linear relationship between FGF23 and stroke, and HF and all-cause mortality. In contrast, FGF23 levels and cardiovascular mortality exhibited a potentially linear relationship. To our knowledge, this is the first dose-response meta-analysis focusing on the association between FGF23 levels and cardiovascular diseases in general populations.

The findings were confirmed with a further analysis stratified by age (≤60 years vs. >60 years), follow-up duration (≤10 years vs. >10 years) and FGF23 types (iFGF23 vs. cGFG23). The results revealed a similar relationship between FGF23 and cardiovascular and all-cause mortality. Notably, most subgroups in this study did not exhibit heterogeneity, and no substantial changes were observed in the pooled *RR* when individual studies were deleted. However, moderate heterogeneity was found in all-cause mortality (*I*^2^ = 49%), and a stratified analysis was performed to explore the sources of heterogeneity. No substantial heterogeneity was detected in the intact FGF23 subgroup. The differences in variable adjustment across studies might lead to an inaccurate estimation of the effect size. Although our analysis showed moderate heterogeneity, a robust association was observed between FGF23 levels and all-cause mortality.

Gao and co-workers (10) reported that elevated FGF23 levels were related to all-cause mortality (*RR*: 1.25, 95%*CI*: 1.14−1.37) and CVDs (*RR*: 1.21, 95%*CI*: 1.13−1.39) in hemodialysis patients. These significant associations support the predictive role of FGF23 in CKD patients. Remarkably, most of the included studies had adjusted for potential confounders, including age, estimated glomerular filtration rate and other risk factors. Consequently, a significant association was observed between FGF23 levels and CVD risk and mortality. Three cohorts (24, 37, 44) did not adjust for CKD or kidney function, which were considered crucial variables in mediating the impact of FGF23 on cardiovascular disease. Nevertheless, higher FGF23 levels were still associated with CVDs and mortality after deleting the eGFR-unadjusted studies (MI *RR*: 1.62, 95%*CI*: 1.07−2.45; stroke *RR:* 1.24, 95%*CI:* 1.03−1.49; HF *RR:* 1.37, 95%*CI:* 1.23−1.53; CVD events *RR:* 1.32, 95%*CI:* 0.93−1.88; cardiovascular mortality *RR:* 1.50, 95%*CI:* 1.30−1.74; all-cause mortality *RR:* 1.50, 95%*CI:* 1.24−1.80). Collectively, our results provided compelling evidence for the close relationship of FGF23 levels with CVDs and mortality in the general population, independent of CKD status. These findings suggest that FGF23 may potentially be applied to predict the risk and mortality of CVDs, irrespective of kidney function.

Age is another vital confounding factor, and its effects on the study results should be explored as it is a well-known traditional risk factor. In most populations, the incidence of CVD increases with age. It is believed that the association between age and CVD reflects metabolic risk factors, such as elevated blood pressure, cholesterol, and diabetes (52). Moreover, two major changes with advancing age are large elastic artery stiffening and endothelial dysfunction, contributing to the development of CVD in the elderly (53). Despite adjusting for those factors, the presence of residual confounding factors such as Klotho cannot be excluded. A close association between FGF23 and klotho levels has been established. FGF23 exerts its biological effects by activating FGFRs, which are dependent on the αKlotho co-receptor. Although klotho is absent in the heart, *in vivo* experiments by Hu et al. (54) showed that high FGF23 concentrations induced direct cardiac toxicity in a klotho-deficient state. Serum and urinary Klotho levels are dramatically decreased during early CKD, while FGF23 levels are increased. Klotho deficiency is a pathogenic factor of CKD progression and CVD. Marcais et al. (55) suggested that evaluating FGF23 in the absence of Klotho data may overemphasize its adverse effects. Unfortunately, most studies did not consider the effect of klotho and did not conduct a separate classification analysis of klotho.

Based on the current evidence, high FGF23 levels are associated with increased risks of CVDs and mortality in the general population. Experimental data in CKD and general populations showed that FGF23 exerts direct cardiac and vascular toxicity, mediating cardiac hypertrophy, cardiac fibrosis, cardiac dysfunction, and diffuse vascular calcification by activating specific myocardial FGF receptors (FGFR) (56). Faul et al. (57) reported that injecting recombinant FGF23 into the myocardium of mice resulted in LVH, inducing a significant increase in heart weight, left ventricular wall thickness, and cross-sectional surface area of individual cardiomyocytes. Previous studies have suggested that FGF23 is associated with vascular endothelial dysfunction, arterial stiffness, and diffuse vascular calcification. In addition, the ERK1/2 signaling pathway may play an essential role in vascular calcification (58). Furthermore, FGF23 exerts indirect adverse cardiac effects, such as regulating sodium retention and excretion in the distal renal tubules, increasing the activation of the renin-angiotensin system, and the production of inflammation and oxidative stress markers (59, 60). However, the predictive effects of FGF23 remains to be demonstrated.

### Comparisons with previous studies

Up to now, several meta-analyses have explored the relationship between FGF23 and CVDs (10–13). The predictive value of FGF23 in CKD patients has been extensively summarized. Cheng et al. (13) concluded that high FGF23 levels were related to all-cause mortality (*RR* 1.46, 95% *CI* 1.38−1.55, *p* < 0.001), CVD (*RR* 1.37, 95% *CI* 1.15−1.63, *p* < 0.001) and renal events (*RR* 1.31, 95% *CI* 1.07−1.59, *p* = 0.008) in pre-dialysis CKD patients. Marthi et al. (11) described the association between FGF23 levels and CVDs in the general population, but did not perform a dose-response analysis. Our study extends previous findings and further clarifies the potential dose-response association between FGF23 and CVD risk and mortality in the general population.

### Policy implications and further research

Theoretically, FGF23 may be applied in the identification of high-risk individuals and could be a novel target to reduce the incidence of cardiovascular events. Phosphate binders, FGF23 antibodies, and FGFR blockers are currently the key therapeutic options. Studies have proposed that circulating FGF23 levels are related to dietary phosphate (Pi) intake levels in healthy people. Consequently, reducing the absorption of dietary phosphate can hypothetically decrease the circulating FGF23 concentrations. Commonly used phosphorus binders include Ca^2+^-containing binders, aluminum-containing binders and non-Ca^2+^ or Ca^2+^-free phosphate binders. However, reducing the absorption of dietary phosphate by phosphate binders or combination therapy only results in modest decreases in FGF23 levels and yields short-lived effects. Whether this is caused by increased intestinal total phosphorus absorption or medication resistance is unclear (61).

The mechanisms regulating FGF23 synthesis are poorly understood. Blocking the main FGFR isoform FGFR4 may reduce the cardiotoxic effects of FGF23, but does not affect its physiological functions. The safety of this method in cardiovascular diseases has already been demonstrated in clinical trials. Conversely, FGF23 antibodies might cause greater side effects than clinical benefits in patients with renal dysfunction (56). Optimally, low FGF23 levels should be maintained while blocking the non-target effects, as opposed to completely depleting it (62).

Traditional biomarkers such as troponin I and T have been widely used in clinical practice for the diagnosis of MI. FGF23, as a novel candidate biomarker of cardiovascular risk, is positively correlated with classical biomarkers of cardiac damage but does not directly depend on them (56). In addition, the combination of these biomarkers has been shown to have a significantly higher predictive value for cardiovascular risk assessment than individually (56). The ankle–brachial index (ABI) can be used to predict the risk of CVD and CHD events and is inexpensive, easily accessible, and non-invasive. However, its sensitivity and specificity still need to be explored (63). The coronary artery calcium score reflects the load of coronary calcification and the degree of coronary atherosclerosis. It is measured by cardiac computer tomography and requires patients to be exposed to ionizing radiation, which is particularly unpopular among young subjects, especially women. This technique is more time-consuming and is also limited by its relatively high cost (64). In contrast, FGF23 concentrations are easily obtainable from the patients’ serum at a low cost, which could prove particularly valuable in emergency situations. Therefore, FGF23 can be used as an early and complementary predictor of adverse cardiac events.

Additionally, FGF23 may potentially predict the prognosis of cardiovascular diseases. Song et al. (65) have reported that FGF23 can independently predict the risk of in-stent restenosis in coronary heart disease patients who underwent PCI with a drug-eluting stent. Cornelissen et al. (66) showed similar accuracy in prognosis estimation between assessing FGF23 levels and the well-established Seattle Heart Failure (SHF) model in patients hospitalized for acute HF. Further understanding of the molecular mechanisms of FGF23 in the cardiovascular system will assist in developing and implementing new therapeutic strategies and prognosis estimation.

### Strength and limitations

This review only included prospective studies, avoiding recall bias. Most of the included studies had a large sample size and had adjusted for potential confounding factors, such as age, gender, race, smoking, BMI, and basic disease histories. Nevertheless, the limitations of this study should be acknowledged. Firstly, our results were based on observational studies and a causal relationship cannot be confirmed. The residual confounding factors and the unmeasured factors could not be ruled out completely due to the inherent nature of observational research. Secondly, the majority of the included studies were performed in the United States or Europe, and the applicability of our findings to the Asian population requires further research. Finally, some studies may have included CKD patients in the general population, affecting the reliability of our results.

## Conclusion

Overall, the increased serum FGF23 levels were associated with increased risks of CVDs and mortality in general populations. There was a potentially non-linear relationship between FGF23 and stroke, HF and all-cause mortality, whereas a potentially linear relationship between FGF23 and cardiovascular mortality was observed. Additional studies are needed to clarify the mechanism between FGF23 and CVDs in the general population. The clinical application of FGF23 levels to predict CVD risk requires further research.

## Data availability statement

The original contributions presented in this study are included in the article/Supplementary material, further inquiries can be directed to the corresponding authors.

## Author contributions

XL and PY were responsible for the entire project and revised the draft. PX, YZ, and ML performed the data extraction and statistical analysis, drafted the first version of the manuscript, and interpreted the data. All authors participated in the interpretation of the results and prepared the final version of the manuscript.
