# Supplementary material for: Fibroblast growth factor-23 and the risk of cardiovascular diseases and mortality in the general population: A systematic review and dose-response meta-analysis
Source: Front Cardiovasc Med. 2022 Nov 3;9:989574. doi: 10.3389/fcvm.2022.989574 (PMC9669381; doi:10.3389/fcvm.2022.989574)
Supplement: Supplementary file 1 [file Data_Sheet_1.docx]

**SUPPLEMENTAL MATERIALS**

**Fibroblast Growth Factor-23 and the Risk of Cardiovascular Diseases and mortality in the general population: A systematic review and dose-response meta-analysis**

**Supplemental Table S1**

| **Section and Topic** | **Item #** | **Checklist item** | **Location where item is reported** |
| --- | --- | --- | --- |
| **TITLE** | | |  |
| Title | 1 | Identify the report as a systematic review. | 1 |
| **ABSTRACT** | | |  |
| Abstract | 2 | See the PRISMA 2020 for Abstracts checklist. | 2 |
| **INTRODUCTION** | | |  |
| Rationale | 3 | Describe the rationale for the review in the context of existing knowledge. | 3 |
| Objectives | 4 | Provide an explicit statement of the objective(s) or question(s) the review addresses. | 3 |
| **METHODS** | | |  |
| Eligibility criteria | 5 | Specify the inclusion and exclusion criteria for the review and how studies were grouped for the syntheses. | 4 |
| Information sources | 6 | Specify all databases, registers, websites, organisations, reference lists and other sources searched or consulted to identify studies. Specify the date when each source was last searched or consulted. | 3 |
| Search strategy | 7 | Present the full search strategies for all databases, registers and websites, including any filters and limits used. | 3 |
| Selection process | 8 | Specify the methods used to decide whether a study met the inclusion criteria of the review, including how many reviewers screened each record and each report retrieved, whether they worked independently, and if applicable, details of automation tools used in the process. | 4 |
| Data collection process | 9 | Specify the methods used to collect data from reports, including how many reviewers collected data from each report, whether they worked independently, any processes for obtaining or confirming data from study investigators, and if applicable, details of automation tools used in the process. | 4 |
| Data items | 10a | List and define all outcomes for which data were sought. Specify whether all results that were compatible with each outcome domain in each study were sought (e.g. for all measures, time points, analyses), and if not, the methods used to decide which results to collect. | 4 |
|  | 10b | List and define all other variables for which data were sought (e.g. participant and intervention characteristics, funding sources). Describe any assumptions made about any missing or unclear information. | 4 |
| Study risk of bias assessment | 11 | Specify the methods used to assess risk of bias in the included studies, including details of the tool(s) used, how many reviewers assessed each study and whether they worked independently, and if applicable, details of automation tools used in the process. |  |
| Effect measures | 12 | Specify for each outcome the effect measure(s) (e.g. risk ratio, mean difference) used in the synthesis or presentation of results. | 4 |
| Synthesis methods | 13a | Describe the processes used to decide which studies were eligible for each synthesis (e.g. tabulating the study intervention characteristics and comparing against the planned groups for each synthesis (item #5)). | 4 |
|  | 13b | Describe any methods required to prepare the data for presentation or synthesis, such as handling of missing summary statistics, or data conversions. | 4 |
|  | 13c | Describe any methods used to tabulate or visually display results of individual studies and syntheses. | 4 |
|  | 13d | Describe any methods used to synthesize results and provide a rationale for the choice(s). If meta-analysis was performed, describe the model(s), method(s) to identify the presence and extent of statistical heterogeneity, and software package(s) used. | 4 |
|  | 13e | Describe any methods used to explore possible causes of heterogeneity among study results (e.g. subgroup analysis, meta-regression). | 5 |
|  | 13f | Describe any sensitivity analyses conducted to assess robustness of the synthesized results. | 5 |
| Reporting bias assessment | 14 | Describe any methods used to assess risk of bias due to missing results in a synthesis (arising from reporting biases). |  |
| Certainty assessment | 15 | Describe any methods used to assess certainty (or confidence) in the body of evidence for an outcome. |  |
| **RESULTS** | | |  |
| Study selection | 16a | Describe the results of the search and selection process, from the number of records identified in the search to the number of studies included in the review, ideally using a flow diagram. | 5 |
|  | 16b | Cite studies that might appear to meet the inclusion criteria, but which were excluded, and explain why they were excluded. | 5 |
| Study characteristics | 17 | Cite each included study and present its characteristics. | 5 |
| Risk of bias in studies | 18 | Present assessments of risk of bias for each included study. | 5 |
| Results of individual studies | 19 | For all outcomes, present, for each study: (a) summary statistics for each group (where appropriate) and (b) an effect estimate and its precision (e.g. confidence/credible interval), ideally using structured tables or plots. | 5-7 |
| Results of syntheses | 20a | For each synthesis, briefly summarise the characteristics and risk of bias among contributing studies. | 5-7 |
|  | 20b | Present results of all statistical syntheses conducted. If meta-analysis was done, present for each the summary estimate and its precision (e.g. confidence/credible interval) and measures of statistical heterogeneity. If comparing groups, describe the direction of the effect. | 5-7 |
|  | 20c | Present results of all investigations of possible causes of heterogeneity among study results. | 5-7 |
|  | 20d | Present results of all sensitivity analyses conducted to assess the robustness of the synthesized results. | 7 |
| Reporting biases | 21 | Present assessments of risk of bias due to missing results (arising from reporting biases) for each synthesis assessed. |  |
| Certainty of evidence | 22 | Present assessments of certainty (or confidence) in the body of evidence for each outcome assessed. |  |
| **DISCUSSION** | | |  |
| Discussion | 23a | Provide a general interpretation of the results in the context of other evidence. | 8 |
|  | 23b | Discuss any limitations of the evidence included in the review. | 11 |
|  | 23c | Discuss any limitations of the review processes used. | 11 |
|  | 23d | Discuss implications of the results for practice, policy, and future research. | 9-10 |
| **OTHER INFORMATION** | | |  |
| Registration and protocol | 24a | Provide registration information for the review, including register name and registration number, or state that the review was not registered. | 3 |
|  | 24b | Indicate where the review protocol can be accessed, or state that a protocol was not prepared. | 11 |
|  | 24c | Describe and explain any amendments to information provided at registration or in the protocol. |  |
| Support | 25 | Describe sources of financial or non-financial support for the review, and the role of the funders or sponsors in the review. | 11 |
| Competing interests | 26 | Declare any competing interests of review authors. | 11 |
| Availability of data, code and other materials | 27 | Report which of the following are publicly available and where they can be found: template data collection forms; data extracted from included studies; data used for all analyses; analytic code; any other materials used in the review. | 11 |
|  |  |  |  |

**Supplemental Table S2**

**Dose-response Meta analysis reporting guidelines (G-Dose Checklist)**

| **structure** | **Report items** | **The page number** |
| --- | --- | --- |
| Title |  |  |
| Topic | Report this study as a dose-response meta-analysis | 1 |
|  | Report the types of included studies: such as randomized controlled trials, cohort studies, case-control studies or others | 1 |
| Abstract |  |  |
| Format | According to the structured summary format or the format required by the journal publication | 2 |
| Content | Study objective, population, exposure factors, primary outcome | 2 |
|  | Search database, time limit, and search results | 2 |
|  | The model used in the meta-analysis was used to reflect the association between different exposure levels and outcomes. Such as: restricted cubic spline function, flexible piecewise polynomial function or other trend approximation function | 2 |
|  | Random, fixed, mass effect or IVhet models | 2 |
|  | Main results and conclusions | 2 |
| Introduction | The research background | 3 |
|  | The clinical or public health problem being studied | 3 |
|  | Existing studies, reviews or meta-analyses have shown the understanding of this problem | 3 |
|  | Purpose and Significance | 3 |
| Methods |  |  |
| Reporting guidelines | Clarify the reporting guidelines used | 3 |
| Inclusion and/or exclusion criteria | PICOS principles: Clarify the target population, intervention/exposure (at least 3 levels of exposure, continuous variable), control (controlled study), outcome, and study type | 4 |
| Literature retrieval | Retrieve resources (e.g., database, manual search) | 3 |
|  | Detailed retrieval strategies (e.g., free terms or subject terms, filters, or wildcards) | 3 |
|  | Excluded literature list and reasons; A flow chart is recommended to record all excluded references | 3 |
|  | Any attempt to contact the original author | 3 |
| Data extraction | Basic information of the included studies, such as first author's name, publication year, study type, follow-up period, adjusted variables, etc | 4 |
|  | Data of dose-response meta-analysis, including dose, number of cases and sample size of each layer of exposure, RR of the corresponding reference level of each layer of exposure (corrected by extracting variables as far as possible), and data type (e.g., incidence, cumulative incidence, etc.) | 4 |
|  | Specify the dose calculation method (e.g., median or mean) | 4 |
|  | A standardized method, such as the conversion of different units | 4 |
| Treatment of bias | Report the risk of bias and use the risk of bias assessment form |  |
| Statistical analysis | Models and common reference levels used in dose-response Meta analysis, such as restricted cubic splines, flexible piecewise polynomials or other trend approximation functions | 4 |
|  | Nonlinearity test methods (e.g., Wald test or likelihood ratio test) | 4 |
|  | The measurement method of heterogeneity | 5 |
|  | Random or fixed effects models were used depending on the investigator's objective or the heterogeneity of the study | 4 |
|  | Weighting methods, such as sample size dependent inverse variance method, IVhet method, M-H method, QE method with bias correction function | 4 |
|  | Methods used to calculate the specified dose for each layer of exposure, e.g., median, mean | 4 |
|  | The processing method of missing value | 4 |
|  | Supplementary analyses, such as subgroup analysis, sensitivity analysis, Meta-regression (only used in linear correlation), goodness of fit, etc | 5 |
|  | Detection and treatment of publication bias | 4 |
|  | Software, such as R software, is recommended to calculate the heterogeneity of multiple variables | 5 |
| Result | Report details of the included studies | 5 |
|  | Main results, and provide related graphs or tables | 5-7 |
|  | Results of supplementary analyses, such as subgroup analysis, sensitivity analysis, Meta-regression (only available for linear correlation), goodness of fit, etc | 5-7 |
|  | Detection results and treatment of publication bias | 7 |
| Discuss | The main findings of the study and the level of evidence for the results | 8 |
|  | Explain the results | 8-9 |
|  | Implications for future research | 9-10 |
|  | Advantages and Limitations | 11 |
|  | Conclusion | 11 |
|  | Sources of fundings | 11 |

**Table S3.Detailed description of the search strategy**

| **PubMed** | |
| --- | --- |
| #1 | Cardiovascular Diseases [MeSH Terms] |
| #2 | (Cardiovascular Disease[Title/Abstract]) OR (Disease, Cardiovascular[Title/Abstract]) OR (Diseases, Cardiovascular[Title/Abstract]) OR (Myocardial infarction[Title/Abstract]) OR Stroke[Title/Abstract] OR (Heart failure[Title/Abstract]) OR (Atrial fibrillation[Title/Abstract]) OR (Coronary heart disease[Title/Abstract]) OR (Left ventricular hypertrophy[Title/Abstract]) OR (Hypertension[Title/Abstract]) |
| #3 | #1 OR #2 |
| #4 | fibroblast growth factor-23 [MeSH Terms] |
| #5 | (FGF-23 protein[Title/Abstract]) OR (fibroblast growth factor 23[Title/Abstract]) OR (FGF-23 protein[Title/Abstract]) OR (phosphatonin[Title/Abstract]) OR (tumor-derived hypophosphatemia inducing factor[Title/Abstract]) |
| #6 | #4 OR #6 |
| #7 | #3 AND #6 |
| **Embase** | |
| #1 | Cardiovascular Disease/exp |
| #2 | (Cardiovascular diseases OR Disease, Cardiovascular OR Diseases, Cardiovascular OR Myocardial infarction OR Stroke OR Heart failure OR Atrial fibrillation OR Coronary heart disease OR Left ventricular hypertrophy OR Hypertension):ti,ab,kw |
| #3 | #1 OR #2 |
| #4 | fibroblast growth factor-23/exp |
| #5 | (FGF-23 protein OR fibroblast growth factor 23 OR FGF-23 protein OR phosphatonin OR tumor-derived hypophosphatemia inducing factor):ti,ab,kw |
| #6 | #4 OR #5 |
| #7 | #3 AND #6 |
| **Cochrane** | |
| #1 | MeSH descriptor:[Cardiovascular Diseases]explode all trees |
| #2 | (Cardiovascular diseases OR Disease, Cardiovascular OR Diseases, Cardiovascular OR Myocardial infarction OR Stroke OR Heart failure OR Atrial fibrillation OR Coronary heart disease OR Left ventricular hypertrophy OR Hypertension):ti,ab,kw |
| #3 | #1 OR #2 |
| #4 | MeSH descriptor:[fibroblast growth factor-23]explode all trees |
| #5 | (FGF-23 protein OR fibroblast growth factor 23 OR FGF-23 protein OR phosphatonin OR tumor-derived hypophosphatemia inducing factor):ti,ab,kw |
| #6 | #4 OR #5 |
| #7 | #3 AND #6 |

**Table S4:** Studies excluded (n=58) with reasons

| **Studies excluded** | **Reasons** |
| --- | --- |
| Mitsnefes,2018^1^ | Pediatric patients |
| Lin,2019^2^ | Pediatric patients |
| Ahmed,2021^3^ | Pediatric patients |
| Dalal,2011^4^ | cross-sectional study |
| Gutiérrez,2009^5^ | cross-sectional study |
| Mirza,2009^6^ | cross-sectional study |
| Wright,2016^7^ | cross-sectional study |
| Seiler,2011^8^ | cross-sectional study |
| Palupi-Baroto,2021^9^ | cross-sectional study |
| Nielsen,2019^10^ | cross-sectional study |
| Ahmad,2017^11^ | cross-sectional study |
| Montford,2013^12^ | cross-sectional study |
| Masson,2015^13^ | cross-sectional study |
| Abiola,2022^14^ | cross-sectional study |
| Miyamura,2015^15^ | Case-control study |
| Tanaka,2016^16^ | Case-control study |
| Johnston,2019^17^ | Case-control study |
| Kim,2016^18^ | Case-control study |
| von Jeinsen,2019^19^ | Case-control study |
| Taylor,2011^20^ | Case-control study |
| Svensson,2022^21^ | Case-control study |
| Akhabue,2019^22^ | Not the target population: hypertension population |
| Ter Maaten,2018^23^ | Not the target population: heart failure population |
| Koller,2015^24^ | Not the target population: heart failure population |
| Frimodt-Møller,2018^25^ | Not the target population: Type 2 Diabetic population |
| Bergmark,2018^26^ | Not the target population: Acute Coronary Syndrome patients |
| Fuernau,2014^27^ | Not the target population: Cardiogenic shock patients |
| Lyngbakken,2018^28^ | Not the target population: acute dyspnea patients |
| Bouma-de Krijger,2014^29^ | Not the target population: Nondialyzed CKD population |
| Baia,2013^30^ | Not the target population: Nondialyzed CKD population |
| Isakova, 2011^31^ | Not the target population: Nondialyzed CKD population |
| Kendrick, 2011^32^ | Not the target population: Nondialyzed CKD population |
| Levin,2014^33^ | Not the target population: Nondialyzed CKD population |
| Scialla,2014^34^ | Not the target population: Nondialyzed CKD population |
| Munoz-Mendoza,2017^35^ | Not the target population: Nondialyzed CKD population |
| Ginsberg,2018^36^ | Not the target population: Nondialyzed CKD population |
| Emrich,2019^37^ | Not the target population: Nondialyzed CKD population |
| Jialal, 2017^38^ | Not the target population: Nondialyzed CKD population |
| Mehta, 2016^39^ | Not the target population: Nondialyzed CKD population |
| Silval, 2019^40^ | Not the target population: Nondialyzed CKD population |
| Chan,2018^41^ | Not the target population: Nondialyzed CKD population |
| Alderson,2016^42^ | Not the target population: Nondialyzed CKD population |
| Chonchol,2015^43^ | Not the target population: Dialysis population |
| Nowak, 2014^44^ | Not the target population: Dialysis population |
| Olauson, 2010^45^ | Not the target population: Dialysis population |
| Scialla, 2015^46^ | Not the target population: Dialysis population |
| Sugimoto, 2014^47^ | Not the target population: Dialysis population |
| Komaba, 2020^48^ | Not the target population: Dialysis population |
| Jean,2009^49^ | Not the target population: Dialysis population |
| Block,2020^50^ | Not the target population: Dialysis population |
| Udell,2014^51^ | Moderate-to-high risk of bias |
| Brandenburg,2015^52^ | Overlapping study |
| Moe,2015^53^ | The outcome data is not complete |
| Nakano,2012^54^ | The outcome data is not complete |
| Seiler,2010^55^ | The outcome data is not complete |
| Seiler,2014^56^ | The outcome data is not complete |
| Plischke,2012^57^ | The outcome data is not complete |
| Bergmark,2019^58^ | The outcome data is not complete |

1. Mitsnefes MM, Betoko A, Schneider MF, et al. FGF23 and left ventricular hypertrophy in children with CKD. *Clinical Journal of the American Society of Nephrology.* 2018;13(1):45-52.

2. Lin Y, Shi L, Liu YY, et al. Relationship between plasma fibroblast growth factor-23 and subclinical cardiovascular damages in children with primary hypertension. *Zhonghua er ke za zhi = Chinese journal of pediatrics.* 2019;57(6):471-476.

3. Ahmed E, El-Sharawy SA, Abdelaziz LR, Khalifa NA. Correlation between fibroblast growth factor 23 and heart failure in children with congenital heart disease. *European Journal of Molecular and Clinical Medicine.* 2021;8(3):3915-3927.

4. Dalal M, Sun K, Cappola AR, et al. Relationship of serum fibroblast growth factor 23 with cardiovascular disease in older community-dwelling women. *European Journal of Endocrinology.* 2011;165(5):797-803.

5. Gutiérrez OM, Januzzi JL, Isakova T, et al. Fibroblast growth factor 23 and left ventricular hypertrophy in chronic kidney disease. *Circulation.* 2009;119(19):2545-2552.

6. Mirza MAI, Larsson A, Melhus H, Lind L, Larsson TE. Serum intact FGF23 associate with left ventricular mass, hypertrophy and geometry in an elderly population. *Atherosclerosis.* 2009;207(2):546-551.

7. Wright CB, Shah NH, Mendez AJ, et al. Fibroblast growth factor 23 is associated with subclinical cerebrovascular damage: The northern manhattan study. *Stroke.* 2016;47(4):923-928.

8. Seiler S, Cremers B, Rebling NM, et al. The phosphatonin fibroblast growth factor 23 links calciumphosphate metabolism with left-ventricular dysfunction and atrial fibrillation. *European Heart Journal.* 2011;32(21):2688-2696.

9. Palupi-Baroto R, Hermawan K, Murni IK, et al. High fibroblast growth factor 23 as a biomarker for severe cardiac impairment in children with chronic kidney disease: A single tertiary center study. *International Journal of Nephrology and Renovascular Disease.* 2021;14:165-171.

10. Nielsen TL, Plesner LL, Warming PE, Mortensen OH, Iversen KK, Heaf JG. FGF23 in hemodialysis patients is associated with left ventricular hypertrophy and reduced ejection fraction. *Nefrologia.* 2019;39(3):258-268.

11. Ahmad FS, Cai X, Kunkel K, et al. Racial/ethnic differences in left ventricular structure and function in chronic kidney disease: The chronic renal insufficiency cohort. *American Journal of Hypertension.* 2017;30(8):822-829.

12. Montford JR, Chonchol M, Cheung AK, et al. Low body mass index and dyslipidemia in dialysis patients linked to elevated plasma fibroblast growth factor 23. *American journal of nephrology.* 2013;37(3):183-190.

13. Masson S, Agabiti N, Vago T, et al. The fibroblast growth factor-23 and Vitamin D emerge as nontraditional risk factors and may affect cardiovascular risk. *Journal of Internal Medicine.* 2015;277(3):318-330.

14. Abiola BI, Raji YR, Ajayi S, et al. Comparative analysis of fibroblast growth Factor-23 as a correlate of cardiovascular disease among individuals with chronic kidney disease, hypertensives, and healthy controls. *Nigerian journal of clinical practice.* 2022;25(8):1247-1255.

15. Miyamura M, Fujita SI, Morita H, et al. Circulating fibroblast growth factor 23 has a U-shaped association with atrial fibrillation prevalence. *Circulation Journal.* 2015;79(8):1742-1748.

16. Tanaka S, Fujita SI, Kizawa S, Morita H, Ishizaka N. Association between FGF23, α-Klotho, and cardiac abnormalities among patients with various chronic kidney disease stages. *PLoS ONE.* 2016;11(7).

17. Johnston JA, Nelson DR, Zhang L, Curtis SE, Voelker JR, Wetterau JR. Estimating the distribution of a novel clinical biomarker (FGF-23) in the US population using findings from a regional research registry. *PLoS One.* 2019;14(6):e0218435.

18. Kim HJ, Park M, Park HC, et al. Baseline FGF23 is associated with cardiovascular outcome in incident PD patients. *Peritoneal Dialysis International.* 2016;36(1):26-32.

19. von Jeinsen B, Sopova K, Palapies L, et al. Bone marrow and plasma FGF-23 in heart failure patients: novel insights into the heart–bone axis. *ESC Heart Failure.* 2019;6(3):536-544.

20. Taylor EN, Rimm EB, Stampfer MJ, Curhan GC. Plasma fibroblast growth factor 23, parathyroid hormone, phosphorus, and risk of coronary heart disease. *American heart journal.* 2011;161(5):956-962.

21. Svensson EH, Söderholm M. Fibroblast growth factor 23 is associated with risk of intracerebral hemorrhage. *European journal of neurology.* 2022;29(1):114-120.

22. Akhabue E, Vu THT, Vaidya A, et al. Fibroblast Growth Factor-23, Heart Failure Risk, and Renin-Angiotensin-Aldosterone-System Blockade in Hypertension: The MESA Study. *American Journal of Hypertension.* 2019;32(1):18-25.

23. ter Maaten JM, Voors AA, Damman K, et al. Fibroblast growth factor 23 is related to profiles indicating volume overload, poor therapy optimization and prognosis in patients with new-onset and worsening heart failure. *International Journal of Cardiology.* 2018;253:84-90.

24. Koller L, Kleber ME, Brandenburg VM, et al. Fibroblast Growth Factor 23 Is an Independent and Specific Predictor of Mortality in Patients With Heart Failure and Reduced Ejection Fraction. *Circulation: Heart Failure.* 2015;8(6):1059-1067.

25. Frimodt-Møller M, Von Scholten BJ, Reinhard H, et al. Growth differentiation factor-15 and fibroblast growth factor-23 are associated with mortality in type 2 diabetes – An observational follow-up study. *PLoS ONE.* 2018;13(4).

26. Bergmark B, Udell J, Morrow D, et al. Association of Fibroblast Growth Factor 23 With Recurrent Cardiovascular Events in Patients After an Acute Coronary Syndrome: a Secondary Analysis of a Randomized Clinical Trial. *JAMA cardiology.* 2018;3(6):473‐480. <https://www.cochranelibrary.com/central/doi/10.1002/central/CN-01618799/full>.

27. Fuernau G, Pöss J, Denks D, et al. Fibroblast growth factor 23 in acute myocardial infarction complicated by cardiogenic shock: A biomarker substudy of the Intraaortic Balloon Pump in Cardiogenic Shock II (IABP-SHOCK II) trial. *Critical Care.* 2014;18(1).

28. Lyngbakken MN, Pervez MO, Brynildsen J, et al. Fibroblast growth factor 23 in patients with acute dyspnea: Data from the Akershus Cardiac Examination (ACE) 2 Study. *Clinical Biochemistry.* 2018;52:41-47.

29. Bouma-de KA, Bots M, Vervloet M, et al. Time-averaged level of fibroblast growth factor-23 and clinical events in chronic kidney disease. *Nephrology, dialysis, transplantation.* 2014;29(1):88‐97. <https://www.cochranelibrary.com/central/doi/10.1002/central/CN-01120447/full>.

30. Baia LC, Humalda JK, Vervloet MG, Navis G, Bakker SJ, de Borst MH. Fibroblast growth factor 23 and cardiovascular mortality after kidney transplantation. *Clin J Am Soc Nephrol.* 2013;8(11):1968-1978.

31. Isakova T, Xie H, Yang W, et al. Fibroblast growth factor 23 and risks of mortality and end-stage renal disease in patients with chronic kidney disease. *Jama.* 2011;305(23):2432-2439.

32. Kendrick J, Cheung AK, Kaufman JS, et al. FGF-23 associates with death, cardiovascular events, and initiation of chronic dialysis. *Journal of the American Society of Nephrology.* 2011;22(10):1913-1922.

33. Levin A, Rigatto C, Barrett B, et al. Biomarkers of inflammation, fibrosis, cardiac stretch and injury predict death but not renal replacement therapy at 1 year in a Canadian chronic kidney disease cohort. *Nephrology Dialysis Transplantation.* 2014;29(5):1037-1047.

34. Scialla JJ, Xie H, Rahman M, et al. Fibroblast growth factor-23 and cardiovascular events in CKD. *Journal of the American Society of Nephrology.* 2014;25(2):349-360.

35. Munoz Mendoza J, Isakova T, Cai X, et al. Inflammation and elevated levels of fibroblast growth factor 23 are independent risk factors for death in chronic kidney disease. *Kidney International.* 2017;91(3):711-719.

36. Ginsberg C, Craven TE, Chonchol MB, et al. PTH, FGF23, and intensive blood pressure lowering in chronic kidney disease participants in SPRINT. *Clinical Journal of the American Society of Nephrology.* 2018;13(12):1816-1824.

37. Emrich IE, Brandenburg V, Sellier AB, et al. Strength of Fibroblast Growth Factor 23 as a Cardiovascular Risk Predictor in Chronic Kidney Disease Weaken by ProBNP Adjustment. *American journal of nephrology.* 2019;49(3):203-211.

38. Jialal I, Camacho F, Nathoo B, Tam P, Pahwa R, Wu GG. Fibroblast Growth Factor 23 Predicts Mortality and End-Stage Renal Disease in a Canadian Asian Population with Chronic Kidney Disease. *Nephron.* 2017;137(3):190-196.

39. Mehta R, Cai X, Lee J, et al. Association of fibroblast growth factor 23 With atrial fibrillation in chronic kidney disease, from the Chronic Renal Insufficiency Cohort Study. *JAMA Cardiology.* 2016;1(5):548-556.

40. Silva AP, Mendes F, Carias E, et al. Plasmatic klotho and fgf23 levels as biomarkers of ckd-associated cardiac disease in type 2 diabetic patients. *International Journal of Molecular Sciences.* 2019;20(7).

41. Chan GC, Divers J, Russell GB, et al. FGF23 concentration and APOL1 genotype are novel predictors of mortality in African Americans with type 2 diabetes. *Diabetes Care.* 2018;41(1):178-186.

42. Alderson HV, Ritchie JP, Middleton R, Larsson A, Larsson TE, Kalra PA. FGF-23 and Osteoprotegerin but not Fetuin-A are associated with death and enhance risk prediction in non-dialysis chronic kidney disease stages 3–5. *Nephrology.* 2016;21(7):566-573.

43. Chonchol M, Greene T, Zhang Y, Hoofnagle A, Cheung A. Low Vitamin D and High Fibroblast Growth Factor 23 Serum Levels Associate with Infectious and Cardiac Deaths in the HEMO Study. *Journal of the American Society of Nephrology : JASN.* 2016;27(1):227‐237. <https://www.cochranelibrary.com/central/doi/10.1002/central/CN-01171019/full>.

44. Nowak A, Friedrich B, Artunc F, et al. Prognostic value and link to atrial fibrillation of soluble klotho and FGF23 in hemodialysis patients. *PLoS ONE.* 2014;9(7).

45. Olauson H, Qureshi AR, Miyamoto T, et al. Relation between serum fibroblast growth factor-23 level and mortality in incident dialysis patients: Are gender and cardiovascular disease confounding the relationship? *Nephrology Dialysis Transplantation.* 2010;25(9):3033-3038.

46. Scialla JJ, Parekh RS, Eustace JA, et al. Race, Mineral Homeostasis and Mortality in Patients with End-Stage Renal Disease on Dialysis. *American journal of nephrology.* 2015;42(1):25-34.

47. Sugimoto H, Ogawa T, Iwabuchi Y, Otsuka K, Nitta K. Relationship between serum fibroblast growth factor-23 level and mortality in chronic hemodialysis patients. *International Urology and Nephrology.* 2014;46(1):99-106.

48. Komaba H, Fuller DS, Taniguchi M, et al. Fibroblast Growth Factor 23 and Mortality Among Prevalent Hemodialysis Patients in the Japan Dialysis Outcomes and Practice Patterns Study. *Kidney International Reports.* 2020;5(11):1956-1964.

49. Jean G, Terrat JC, Vanel T, et al. High levels of serum fibroblast growth factor (FGF)-23 are associated with increased mortality in long haemodialysis patients. *Nephrology Dialysis Transplantation.* 2009;24(9):2792-2796.

50. Block G, Chertow G, Cooper K, et al. Fibroblast growth factor 23 as a risk factor for cardiovascular events and mortality in patients in the EVOLVE trial. *Hemodialysis international.* 2020. <https://www.cochranelibrary.com/central/doi/10.1002/central/CN-02194084/full>.

51. Udell J, Morrow D, Jarolim P, et al. Fibroblast growth factor-23, cardiovascular prognosis, and benefit of angiotensin-converting enzyme inhibition in stable ischemic heart disease. *Journal of the American College of Cardiology.* 2014;63(22):2421‐2428. <https://www.cochranelibrary.com/central/doi/10.1002/central/CN-01015391/full>.

52. Brandenburg VM, Kleber ME, Vervloet MG, et al. Soluble klotho and mortality: The Ludwigshafen Risk and Cardiovascular Health Study. *Atherosclerosis.* 2015;242(2):483-489.

53. Moe S, Chertow G, Parfrey P, et al. Cinacalcet, Fibroblast Growth Factor-23, and Cardiovascular Disease in Hemodialysis: the Evaluation of Cinacalcet HCl Therapy to Lower Cardiovascular Events (EVOLVE) Trial. *Circulation.* 2015;132(1):27‐39. <https://www.cochranelibrary.com/central/doi/10.1002/central/CN-01109138/full>.

54. Nakano C, Hamano T, Fujii N, et al. Intact fibroblast growth factor 23 levels predict incident cardiovascular event before but not after the start of dialysis. *Bone.* 2012;50(6):1266-1274.

55. Seiler S, Reichart B, Roth D, Seibert E, Fliser D, Heine GH. FGF-23 and future cardiovascular events in patients with chronic kidney disease before initiation of dialysis treatment. *Nephrology Dialysis Transplantation.* 2010;25(12):3983-3989.

56. Seiler S, Rogacev KS, Roth HJ, et al. Associations of FGF-23 and sklotho with cardiovascular outcomes among patients with CKD stages 2–4. *Clinical Journal of the American Society of Nephrology.* 2014;9(6):1049-1058.

57. Plischke M, Neuhold S, Adlbrecht C, et al. Inorganic phosphate and FGF-23 predict outcome in stable systolic heart failure. *European Journal of Clinical Investigation.* 2012;42(6):649-656.

58. Bergmark B, Udell J, Morrow D, et al. Klotho, fibroblast growth factor-23, and the renin-angiotensin system - an analysis from the PEACE trial. *European journal of heart failure.* 2019;21(4):462‐470. <https://www.cochranelibrary.com/central/doi/10.1002/central/CN-02162767/full>.

**Table S5**. Quality assessment of included studies

| Author  (Publication Year) | NEWCASTLE OTTAWA SCALE (COHORT STUDIES) | | | | | | | | | |
| --- | --- | --- | --- | --- | --- | --- | --- | --- | --- | --- |
|  | Selection | | | Comparability | | | Outcome | | | Total |
|  | a | b | c | d | e | f | g | h | i |  |
| Ärnlöv,2012 | 1 | 1 | 1 | 1 | 1 | 0 | 1 | 1 | 0 | 7 |
| Ärnlöv,2013 | 0 | 1 | 1 | 0 | 1 | 1 | 1 | 1 | 1 | 7 |
| Brandenburg,2014 | 0 | 1 | 1 | 0 | 1 | 1 | 1 | 1 | 1 | 7 |
| Ix,2012 | 1 | 1 | 1 | 1 | 1 | 0 | 1 | 1 | 1 | 8 |
| Kestenbaum,2014 | 0 | 1 | 1 | 1 | 1 | 1 | 1 | 1 | 0 | 7 |
| Lutsey,2014 | 1 | 1 | 1 | 1 | 1 | 1 | 1 | 1 | 0 | 8 |
| Parker,2010 | 0 | 1 | 1 | 1 | 1 | 1 | 1 | 1 | 1 | 8 |
| Westerberg,2013 | 1 | 1 | 1 | 1 | 1 | 1 | 1 | 0 | 0 | 7 |
| Wright,2014 | 1 | 1 | 1 | 1 | 1 | 1 | 1 | 1 | 1 | 9 |
| Souma,2016 | 1 | 1 | 1 | 1 | 1 | 1 | 1 | 1 | 1 | 9 |
| Almahmoud,2018 | 1 | 1 | 1 | 1 | 1 | 1 | 0 | 1 | 1 | 8 |
| De Jong,2021 | 1 | 1 | 1 | 1 | 1 | 1 | 1 | 1 | 1 | 9 |
| Sharma,2021 | 1 | 1 | 1 | 1 | 0 | 1 | 1 | 1 | 1 | 8 |
| Robinson-Cohen,2020 | 1 | 0 | 1 | 1 | 1 | 1 | 1 | 1 | 1 | 8 |
| Haring,2016 | 1 | 1 | 1 | 1 | 1 | 1 | 1 | 1 | 1 | 9 |
| Deo,2015 | 1 | 1 | 1 | 1 | 1 | 0 | 1 | 1 | 0 | 7 |
| Mathew,2014 | 1 | 1 | 1 | 1 | 1 | 1 | 1 | 1 | 0 | 8 |
| Alonso,2014 | 0 | 1 | 1 | 1 | 1 | 1 | 1 | 1 | 1 | 8 |
| Akhabue,2018 | 1 | 1 | 1 | 1 | 1 | 1 | 1 | 0 | 0 | 7 |
| Fyfe-Johnson,2016 | 1 | 1 | 1 | 1 | 1 | 0 | 1 | 1 | 0 | 7 |
| Drew,2020 | 1 | 1 | 1 | 1 | 1 | 0 | 1 | 1 | 1 | 8 |
| Jovanovich,2013 | 0 | 1 | 1 | 1 | 1 | 1 | 1 | 0 | 1 | 7 |
| Binnenmars,2022 | 0 | 1 | 1 | 1 | 1 | 1 | 1 | 1 | 1 | 8 |
| Paul,2021 | 0 | 1 | 1 | 1 | 1 | 1 | 1 | 1 | 1 | 8 |

1. Representativeness of the exposed cohort.
2. Selection of the non-exposed cohort.
3. Ascertainment of exposure.
4. Demonstration that outcome of interest was not present at start of study.
5. Comparability of cohorts on the basis of the design or analysis (adjusted for age).
6. Comparability of cohorts on the basis of the design or analysis (adjusted for any other factor).
7. Assessment of outcome.
8. Was follow-up long enough for outcomes to occur. (>5 years for new on-set, ).
9. Adequacy of follow-up of cohorts.

| Author  (Publication Year) | CASE-COHORT/CASE-CONTROL STUDIES | | | | | | | | | |
| --- | --- | --- | --- | --- | --- | --- | --- | --- | --- | --- |
|  | Selection | | | Comparability | | | Outcome | | | Total |
|  | a | b | c | d | e | f | g | h | i |  |
| Di Giuseppe,2014 | 1 | 1 | 1 | 1 | 1 | 1 | 1 | 1 | 0 | 8 |
| Di Giuseppe,2015 | 1 | 1 | 1 | 0 | 1 | 1 | 1 | 1 | 0 | 7 |
| Panwar,2015 | 1 | 1 | 1 | 1 | 1 | 1 | 1 | 1 | 0 | 8 |
| Sharma,2020 | 1 | 1 | 1 | 1 | 1 | 1 | 1 | 1 | 1 | 9 |
| panwar,2018 | 1 | 1 | 1 | 1 | 1 | 1 | 1 | 0 | 1 | 9 |

a.Is the case definition adequate?

b.Representativeness of the cases

c.Selection of controls

d.Definition of controls

e.Comparability of cases and controls on the basis of the design or analysis (adjusted for age).

f.Comparability of cases and cohorts on the basis of the design or analysis (adjusted for any other factor).

g.Assessment of exposure.

h.Same method of ascertainment for cases and controls.

i.Non-Response rate.

**SUPPLEMENTAL FIGURES**

**
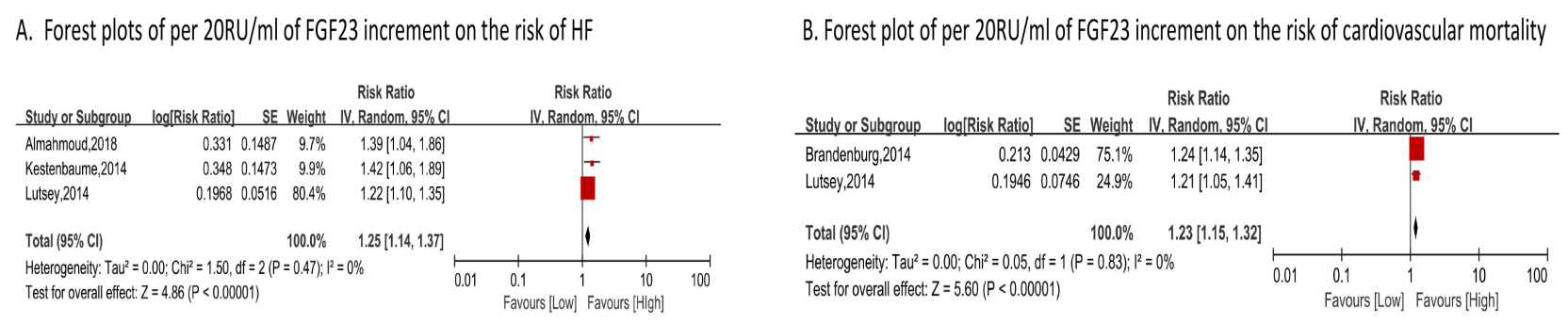
**

**Figure S1. Forest plots of per 20RU/ml of FGF23 increment on the risk of HF and cardiovascular mortality.**


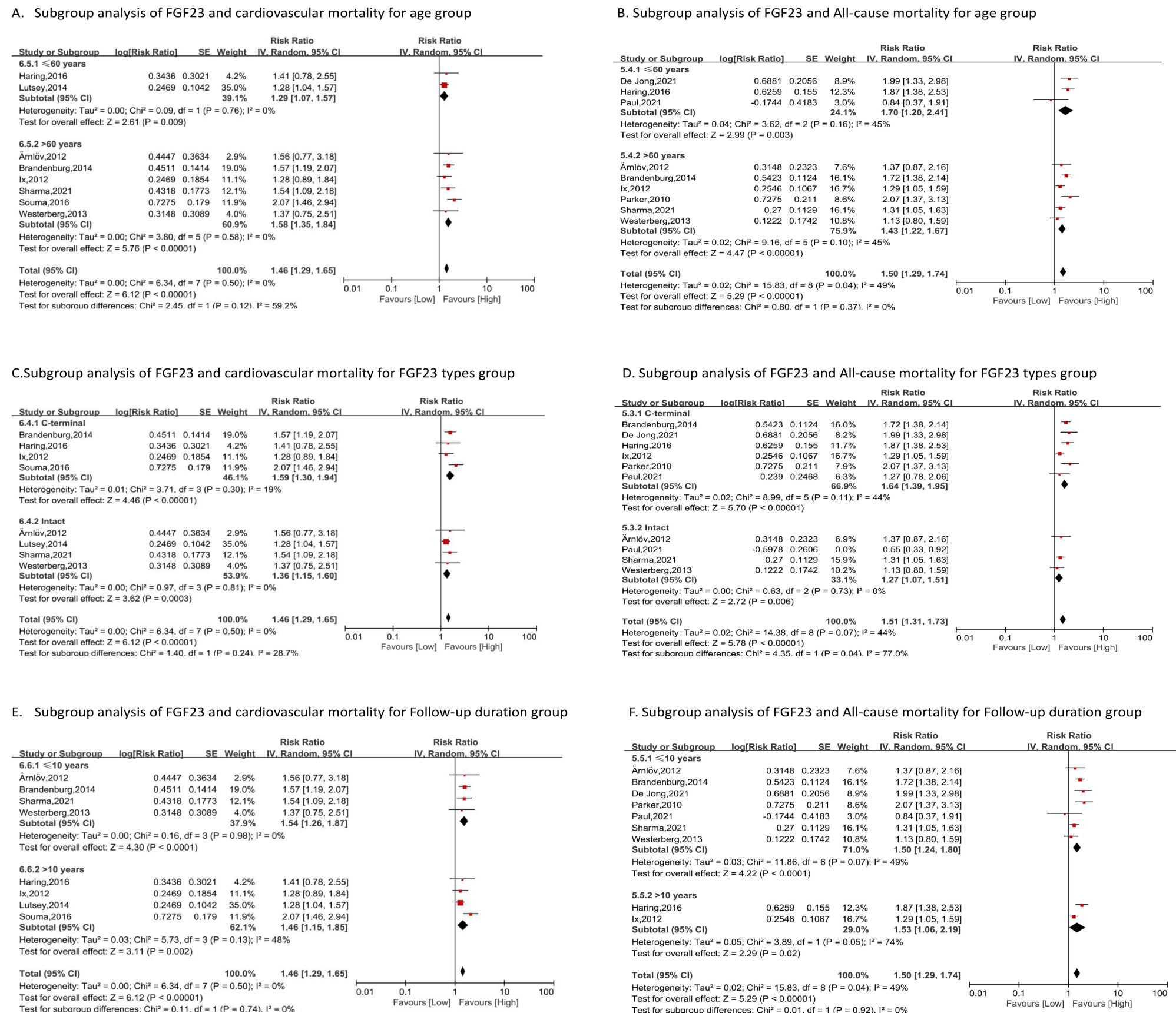


**Figure S2. Subgroups analysis of FGF-23 and cardiovascular mortality and All-cause mortality.**


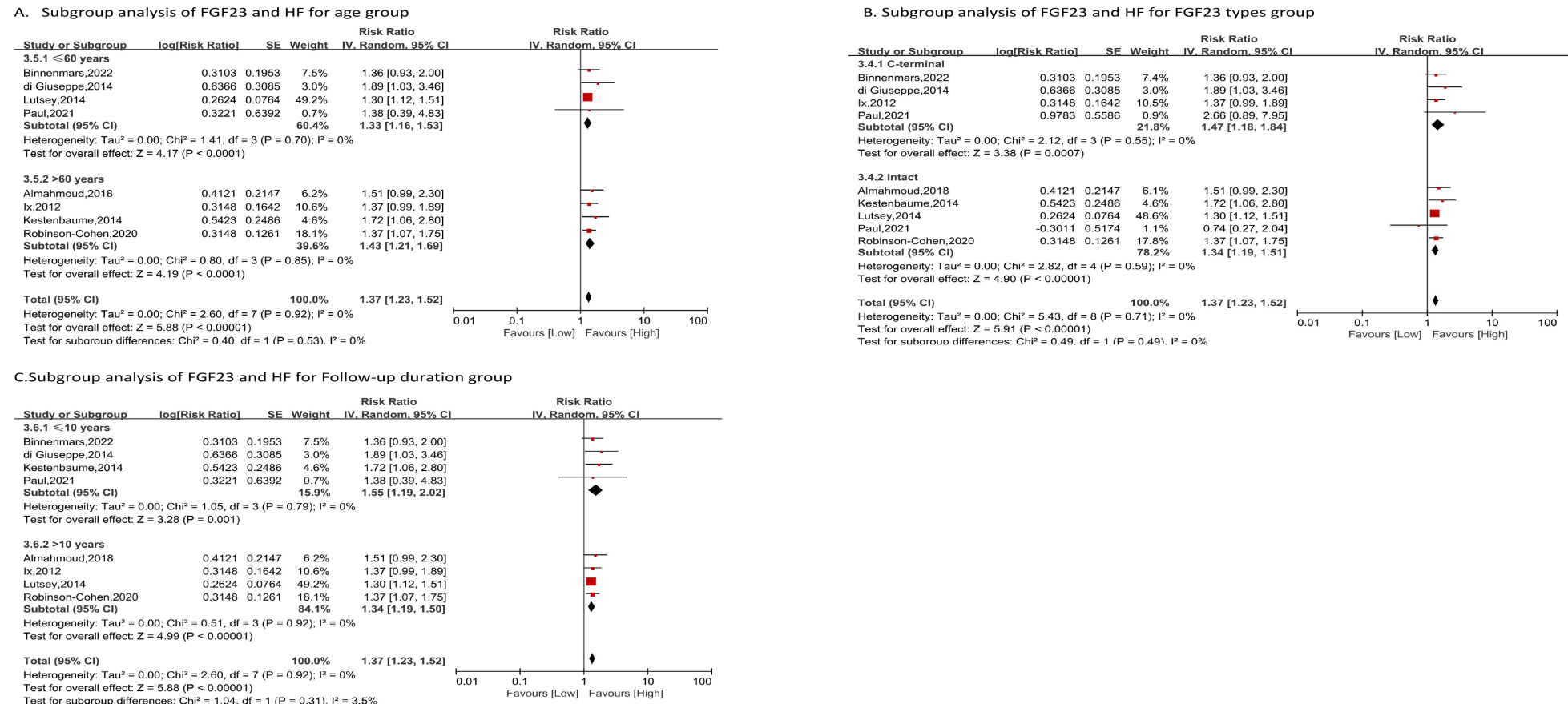


**Figure S3. Subgroups analysis of FGF-23 and HF**

**
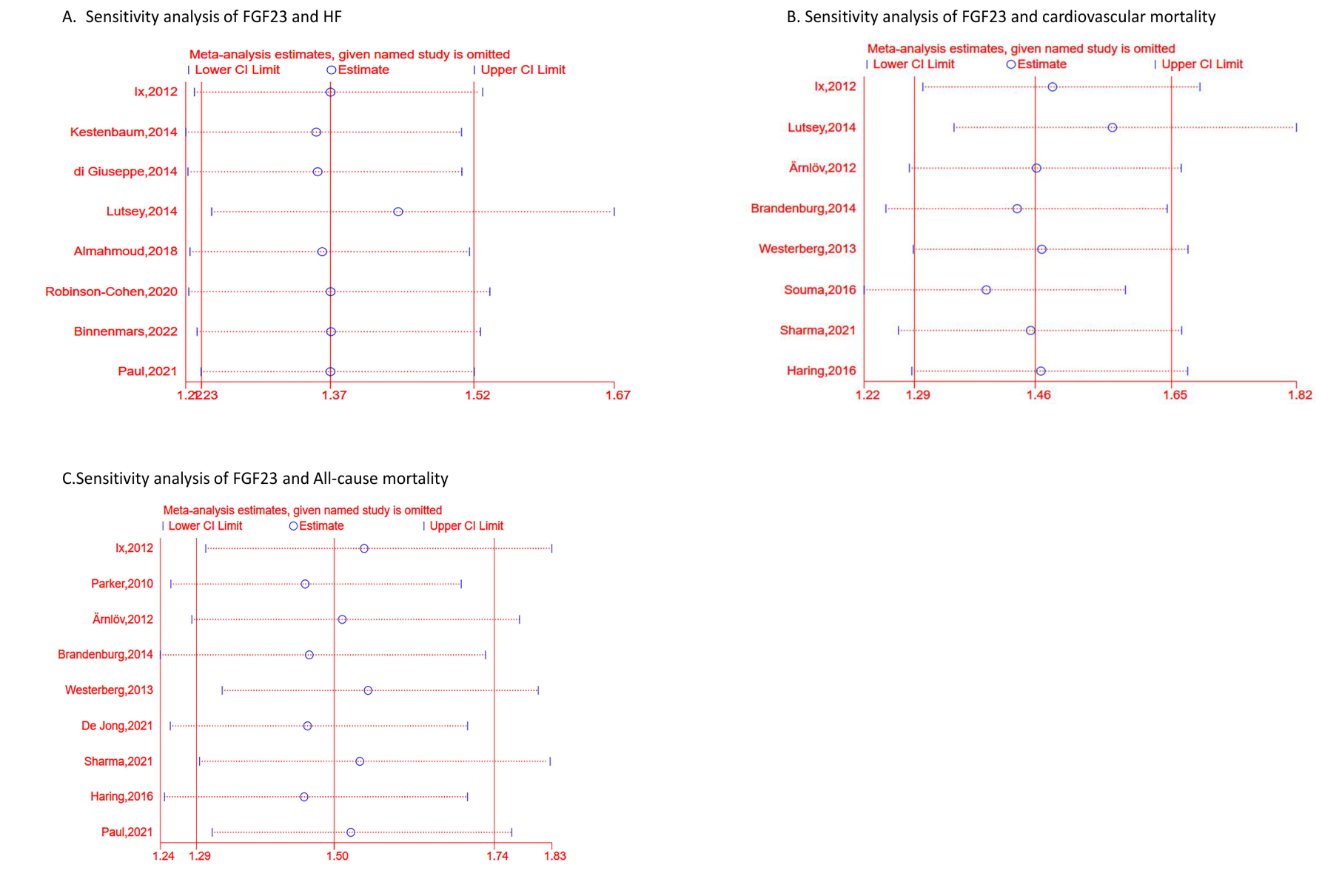
**

**Figure S4. [Sensitivity](javascript:;) [analysis](javascript:;) of FGF23 and HF, cardiovascular mortality and All-cause mortality.**

**
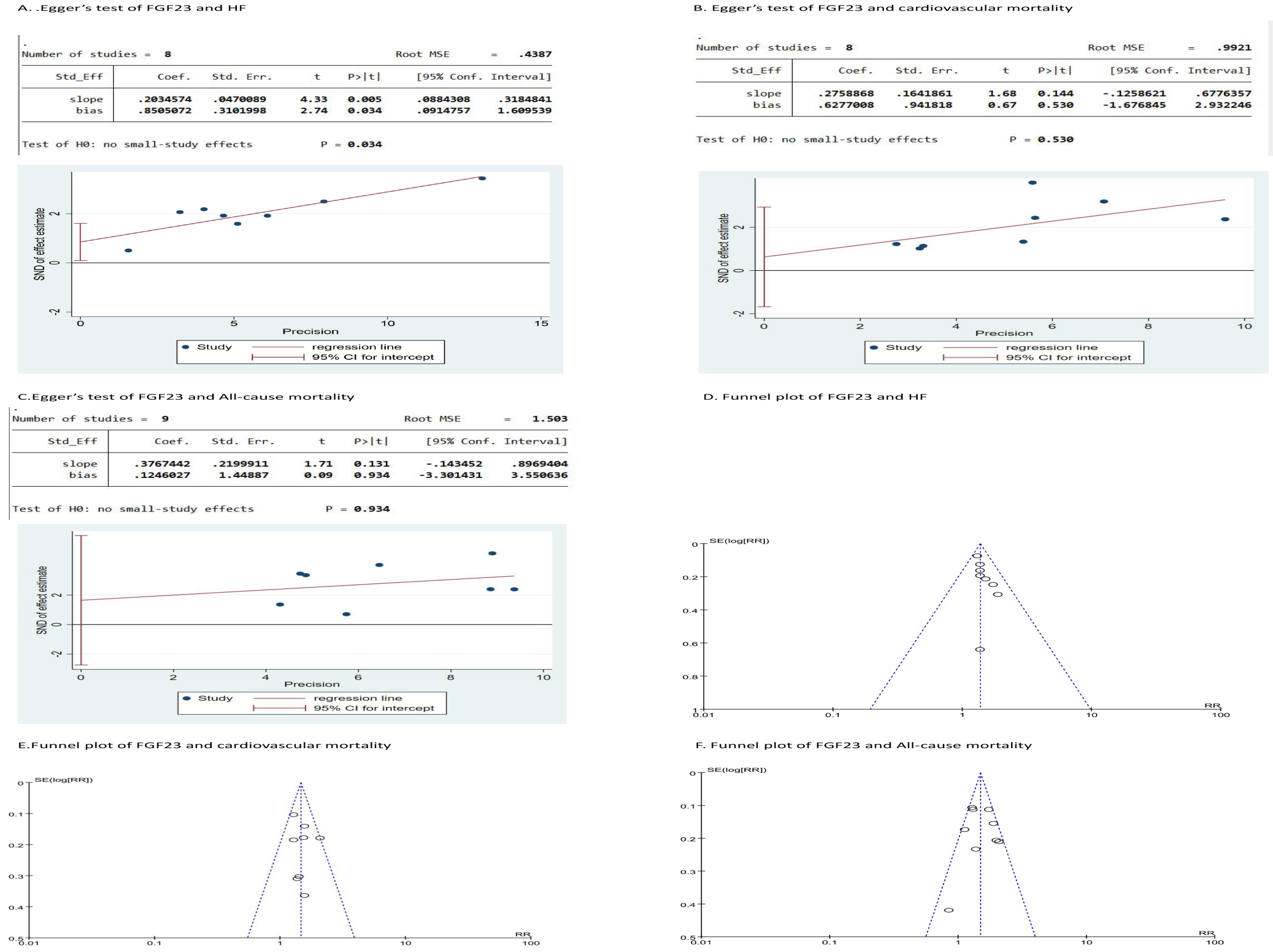
**

**Figure S5. Egger’s test and Funnel plot of FGF-23 and cardiovascular mortality and All-cause mortality.**
