# Supplementary figures and images for: Fibroblast growth factor-23 and the risk of cardiovascular diseases and mortality in the general population: A systematic review and dose-response meta-analysis
Source: Front Cardiovasc Med. 2022 Nov 3;9:989574. doi: 10.3389/fcvm.2022.989574 (PMC9669381; doi:10.3389/fcvm.2022.989574)

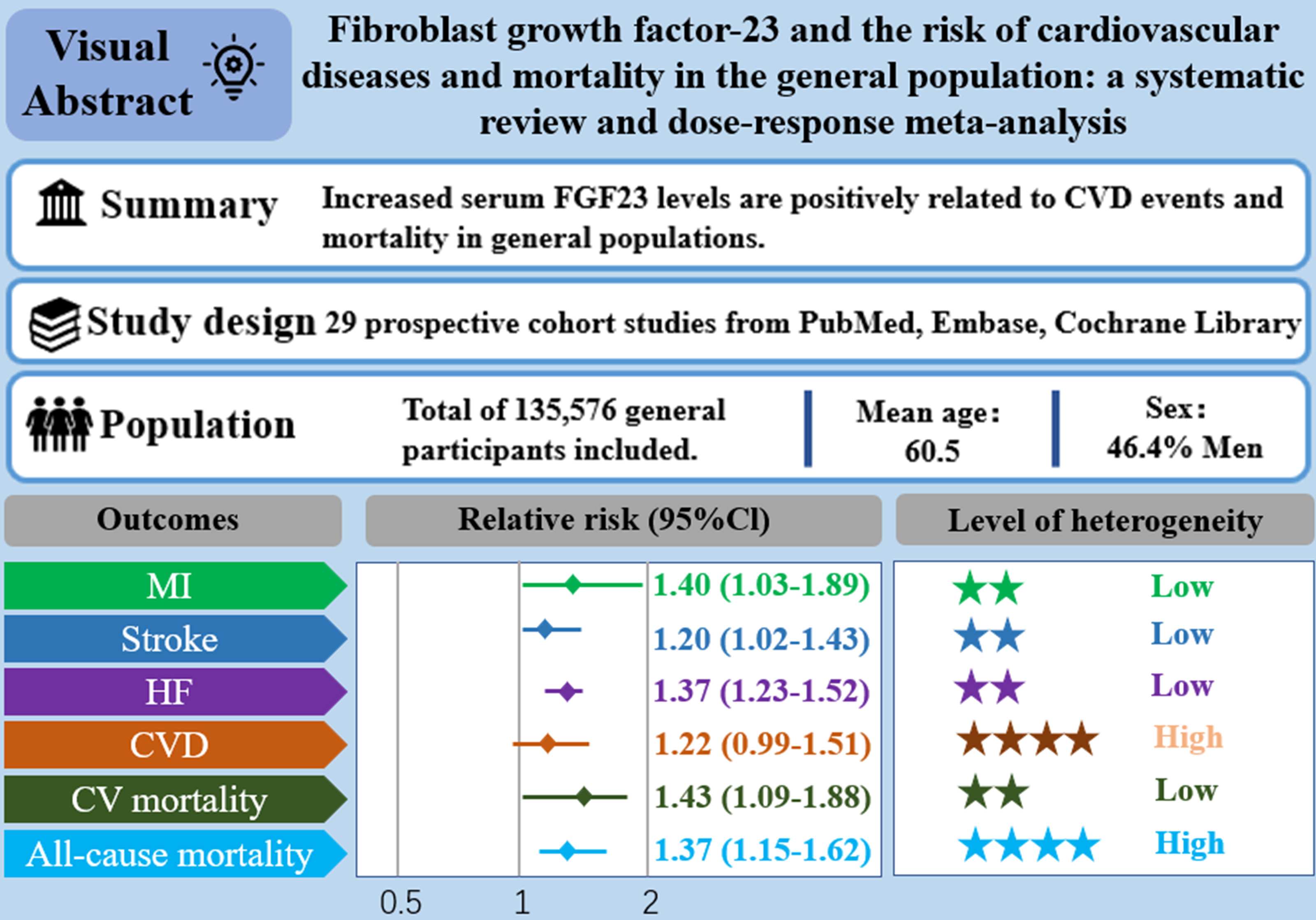

Supplement: Supplementary file 2 [file Image_1.TIF]
